# The personality traits activity, self-reproach, and negative affect jointly predict clinical recurrence, depressive symptoms, and low quality of life in inflammatory bowel disease patients

**DOI:** 10.1007/s00535-022-01902-7

**Published:** 2022-07-28

**Authors:** Sebastian Bruno Ulrich Jordi, Brian Matthew Lang, Jacqueline Wyss, Bianca Auschra, Bahtiyar Yilmaz, Niklas Krupka, Thomas Greuter, Philipp Schreiner, Luc Biedermann, Martin Preisig, Roland von Känel, Gerhard Rogler, Stefan Begré, Benjamin Misselwitz, The Swiss IBD cohort study group, Claudia Anderegg, Peter Bauerfeind, Christoph Beglinger, Stefan Begré, Dominique Belli, José M. Bengoa, Luc Biedermann, Beat Bigler, Janek Binek, Mirjam Blattmann, Stephan Boehm, Jan Borovicka, Christian P. Braegger, Nora Brunner, Patrick Bühr, Bernard Burnand, Emanuel Burri, Sophie Buyse, Matthias Cremer, Dominique H. Criblez, Philippe de Saussure, Lukas Degen, Joakim Delarive, Christopher Doerig, Barbara Dora, Gian Dorta, Mara Egger, Tobias Ehmann, Ali El-Wafa, Matthias Engelmann, Jessica Ezri, Christian Felley, Markus Fliegner, Nicolas Fournier, Montserrat Fraga, Pascal Frei, Remus Frei, Michael Fried, Florian Froehlich, Christian Funk, Raoul Ivano Furlano, Suzanne Gallot-Lavallée, Martin Geyer, Marc Girardin, Delphine Golay, Tanja Grandinetti, Beat Gysi, Horst Haack, Johannes Haarer, Beat Helbling, Peter Hengstler, Denise Herzog, Cyrill Hess, Klaas Heyland, Thomas Hinterleitner, Philippe Hiroz, Claudia Hirschi, Petr Hruz, Rika Iwata, Res Jost, Pascal Juillerat, Vera Kessler Brondolo, Christina Knellwolf, Christoph Knoblauch, Henrik Köhler, Rebekka Koller, Claudia Krieger-Grübel, Gerd Kullak-Ublick, Patrizia Künzler, Markus Landolt, Rupprecht Lange, Frank Serge Lehmann, Andrew Macpherson, Philippe Maerten, Michel H. Maillard, Christine Manser, Michael Manz, Urs Marbet, George Marx, Christoph Matter, Valérie McLin, Rémy Meier, Martina Mendanova, Christa Meyenberger, Pierre Michetti, Benjamin Misselwitz, Darius Moradpour, Bernhard Morell, Patrick Mosler, Christian Mottet, Christoph Müller, Pascal Müller, Beat Müllhaupt, Claudia Münger-Beyeler, Leilla Musso, Andreas Nagy, Michaela Neagu, Cristina Nichita, Jan Niess, Natacha Noël, Andreas Nydegger, Nicole Obialo, Carl Oneta, Cassandra Oropesa, Ueli Peter, Daniel Peternac, Laetitia Marie Petit, Franziska Piccoli-Gfeller, Julia Beatrice Pilz, Valérie Pittet, Nadia Raschle, Ronald Rentsch, Sophie Restellini, Jean-Pierre Richterich, Sylvia Rihs, Marc Alain Ritz, Jocelyn Roduit, Daniela Rogler, Gerhard Rogler, Jean-Benoît Rossel, Markus Sagmeister, Gaby Saner, Bernhard Sauter, Mikael Sawatzki, Michela Schäppi, Michael Scharl, Martin Schelling, Susanne Schibli, Hugo Schlauri, Sybille Schmid Uebelhart, Jean-François Schnegg, Alain Schoepfer, Frank Seibold, Mariam Seirafi, Gian-Marco Semadeni, David Semela, Arne Senning, Marc Sidler, Christiane Sokollik, Johannes Spalinger, Holger Spangenberger, Philippe Stadler, Michael Steuerwald, Alex Straumann, Bigna Straumann-Funk, Michael Sulz, Joël Thorens, Sarah Tiedemann, Radu Tutuian, Stephan Vavricka, Francesco Viani, Jürg Vögtlin, Roland von Känel, Alain Vonlaufen, Dominique Vouillamoz, Rachel Vulliamy, Jürg Wermuth, Helene Werner, Paul Wiesel, Reiner Wiest, Tina Wylie, Jonas Zeitz, Dorothee Zimmermann

**Affiliations:** 1 Department of Visceral Surgery and Medicine, Inselspital, Bern University Hospital, University of Bern, Bern, Switzerland; 2 Department of Gastroenterology and Hepatology, University Hospital Zurich and Zurich University, Zurich, Switzerland; 3 Department for Biomedical Research, Visceral Surgery and Medicine, Systems Biomedicine of Cellular Development and Signaling in Health and Disease, University of Bern, Bern, Switzerland; 4 Clinic for Transplantation Immunology and Nephrology (Swiss Transplant Cohort Study), University Hospital of Basel, Basel, Switzerland; 5 Department of Consultation-Liaison Psychiatry and Psychosomatic Medicine, University Hospital Zurich, University of Zurich, Zurich, Switzerland; 6 Division of Gastroenterology and Hepatology, University Hospital Lausanne-CHUV, Lausanne, Switzerland; 7 Department of Psychiatry, Lausanne University Hospital and University of Lausanne, Lausanne, Switzerland; 8 Neurology, Department of Biomedical Research, Bern University Hospital, University of Bern, Bern, Switzerland; 9 ISFOM-Institute of Stress Diseases and Stressmanagement, Zurich, Switzerland

**Keywords:** IBD, Personality, NEO-FFI, Five-factor model, Flares

## Abstract

**Background:**

The bidirectional “gut-brain axis” has been implicated in the pathogenesis of inflammatory bowel diseases (IBD). While the influence of stress and depressive symptoms on IBD is well-characterized, the role of personality remains insufficiently investigated.

**Methods:**

Personality was assessed in 1154 Swiss IBD cohort study (SIBDCS) patients via the NEO-Five-Factor Inventory (NEO-FFI) as well as in 2600 participants of the population-based CoLaus¦PsyCoLaus cohort study (NEO-FFI-revised). The NEO-FFI subcomponents activity, self-reproach and negative affect were associated with higher IBD disease activity and were combined to a NEO-FFI risk score. This risk score was validated and its effect on clinical IBD course and psychological endpoints was analysed in time-to-event and cumulative incidence analyses.

**Results:**

In time-to-event analyses, a high NEO-FFI risk score was predictive for the clinical endpoints of new extraintestinal manifestation [EIM, adjusted hazard ratio (aHR) = 1.64, corrected *p* value (*q*) = 0.036] and two established composite flare endpoints (aHR = 1.53–1.63, *q* = 0.003–0.006) as well as for the psychological endpoints depressive symptoms (aHR = 7.06, *q* < 0.001) and low quality of life (aHR = 3.06, *q* < 0.001). Furthermore, cumulative incidence analyses showed that patients at high NEO-FFI risk experienced significantly more episodes of active disease, new EIMs, one of the flare endpoints, depressive episodes and low disease-related quality of life. Personalities of IBD patients showed only minor differences from the general population sample (Pearson’s *r* = 0.03–0.14).

**Conclusions:**

Personality assessed by the NEO-FFI contained considerable predictive power for disease recurrence, depressive symptoms and low quality of life in IBD patients. Nevertheless, the personalities of IBD patients did not substantially differ from the general population.

**Supplementary Information:**

The online version contains supplementary material available at 10.1007/s00535-022-01902-7.

## Introduction

Inflammatory bowel diseases (IBD) are immune-mediated diseases characterized by chronic inflammation of the gastrointestinal tract. They include the subtypes Crohn’s disease (CD), ulcerative colitis (UC), and IBD unclassified (IBD-U). In 2017 6.8 million patients suffered from IBD worldwide [1], and the incidence is further increasing especially in newly westernised regions [2]. While the exact cause of IBD remains unknown, the multifactorial pathogenesis likely includes environmental factors, the gut microbiota as well as immunological and genetic parameters [3].

The clinical course of IBD is usually characterized by periods of quiescent disease with intermittent flare-ups of inflammation that are difficult to predict. Frequent and prolonged inflammation often results in structural damage to the gastrointestinal tract and reduced quality of life. Therefore, tools helping physicians to predict individual patients’ disease courses could decisively improve patient care.

The concept of a “gut brain axis” is well established and there is convincing evidence showing complex interactions between the gastrointestinal tract and the brain [4]. Specifically in IBD patients, mental stress has been shown to increase pro-inflammatory cytokines and to negatively influence the disease course via the gut-brain-axis [5]. Besides stress in general, depression and anxiety have also been associated with IBD[6] and both have been shown to be potential triggers of flares [7–9]. In this context, the role of a patient’s personality on IBD course or even pathogenesis has been debated for a long but remains only insufficiently studied.

Nevertheless, research has demonstrated that personality influences the biological response to stress and therefore an individual’s vulnerability to stress [10]. In accordance with this, our group showed recently that Type D (distressed) personality[11] identifies IBD patients at higher risk for depression and flare-ups and can thus help to predict the disease course [12].

According to Gordon W. Allport’s definition, personality comprises the dynamic mental organization within a person, resulting in reoccurring, temporally stable and context-dependent patterns of behaviours and thoughts [13]. Therefore, personality structure describes a complex and multi-dimensional phenomenon exceeding binary constellations such as Type D personality. Multidimensional personality concepts allow to group and compare patients based on similarities and differences in one or several personality dimensions.

The Five-Factor Model (FFM), also known as the Big Five is an internationally established and extensively researched consensual framework to assess personality traits [14]. It comprises the 5 domains Neuroticism, Extraversion, Openness to experience, Agreeableness, and Conscientiousness. The FFM has been widely applied in medical research, demonstrating how personality impacts patients’ health. For example, low Conscientiousness was found to be associated with higher all-cause mortality in a meta-analysis including 76,150 adults[15] while high Openness appears to be an independent protective factor for incident coronary heart disease [16].

Personality might impact the course of IBD due to its strong association with stress and depression[10] which both are predictors of an unfavourable IBD disease course [6–8]. Moreover, in the past, an “IBD personality”, which predisposes to the development of bowel inflammation had been suggested, an idea which has been highly controversial in the following years [17]. We used data on personality traits from the SIBDCS[18] and the population-based CoLaus¦PsyCoLaus study[19] as a comparison group to investigate the role of personality in IBD and to understand what components of personality are relevant for the prediction of IBD disease course of individual patients.

## Methods

### Study concept and design of the Swiss IBD cohort

**Fig. 1:**
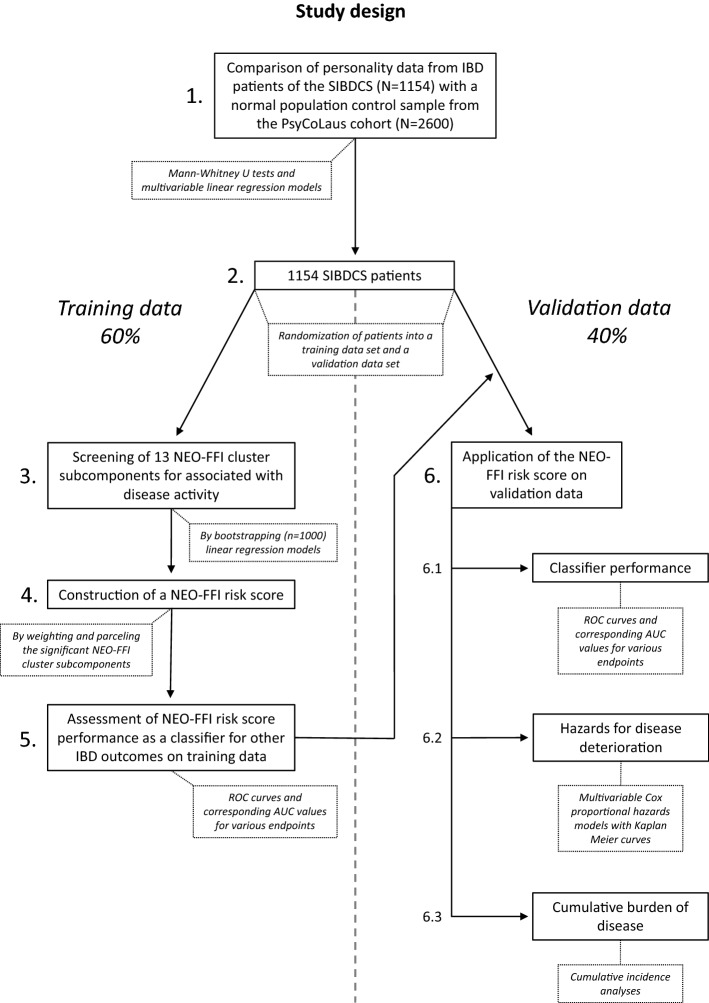
Analysis concept. Flow chart illustrating the order of analysis steps (indicated by grey numbers) and consequent separation of training and validation data (indicated by the vertical dashed line). *IBD* inflammatory bowel disease, *NEO-FFI* NEO five-factor inventory, *SIBDCS* swiss IBD cohort study

The methodology of our analysis strategy partly follows two previous SIBDCS publications (e.g., clinical endpoints) to allow the comparison of results [7, 12]. The study concept and patient selection are illustrated in Fig. 1. We used data from the SIBDCS which is a prospective cohort study with yearly follow-ups [18, 20]. The study has been recruiting IBD patients in Switzerland since 2006. Clinical data are either assessed by physicians or collected directly via patient questionnaires. The SIBDCS’ detailed aims and methodology are described elsewhere [18, 20].

To compare personality findings from the SIBDCS with the normal population, we used population-based data from the CoLaus¦PsyCoLaus study [19]. This single-centre cohort study includes a random sample of residents of the city of Lausanne (Switzerland). The NEO-FFI data used in the present analyses were assessed at the first (2009–2013) and third follow-up (2018–2021).

### SIBDCS patient characteristics

Only SIBDCS patients with NEO-FFI data were included. Various sociodemographic and clinical variables assessed at enrolment and follow-ups were exported: the Crohn’s disease activity index (CDAI) or the modified Truelove and Witts activity index (MTWAI) for patients with CD and UC/IBD-U, respectively, were used to measure disease activity. To obtain intercomparable scales, CDAI and MTWAI scores were *z* score normalized and combined $z{\kern 1pt} = \frac{{{\kern 1pt} x - \overline{x}}}{s}$. Alternatively, we used established cut-off values defining active disease (see below). For definition of complications, extraintestinal manifestations (EIM), stenosis, fistula, surgery, tumour necrosis factor (TNF) inhibitors, number of current therapies, smoking status, physical activity and academic education we exactly adhered to the methodology reported in two earlier SIBDC studies (Supplementary Table 1).[7, 12]

Inclusion into SIBDCS requires the clinical diagnosis of IBD for ≥ 3 months (established by a physician). Therefore, all patients are expected to have had unequivocal signs of intestinal inflammation and gastrointestinal symptoms at least for some time, excluding cases of irritable bowel syndrome which can only be diagnosed in the absence of other gastrointestinal disorders. However, this is not independently confirmed in the SIBDCS.

### Assessment of personality

Research on the FFM led to the development of the NEO Five-Factor Inventory (NEO-FFI) which is one of the most used FFM inventories [21]. Subsequently, more specific item-cluster subcomponents for each of the five broad domains were developed [22]. These 13 narrow subcomponents allow to measure personality at a higher level of detail with reliability (i.e., an average Cronbach’s α of 0.70 in a development sample and 0.66 in a cross-validation sample) comparable to items of established standard FFM inventories such as the NEO-PI-R [23, 24].

Personality data of SIBDCS patients were assessed via the NEO-FFI[21] that consists of 60 items that are answered on a 5-point Likert scale, ranging from strongly disagree to strongly agree (i.e., 0–4 pts.). We applied Saucier’s alternative 1 item coding which uses 58 of 60 items to build 13 cluster subcomponents (for details see Supplementary Table 2) [22, 23]. The two items that are not part of Saucier’s alternative 1 coding (i.e., items number 28 and 33) were not assessed by the SIBDCS. Both items are part of the Openness domain of the traditional 5 main personality domains.

The CoLaus¦PsyCoLaus study assessed participants’ personality with the comparable revised version of the NEO-FFI (NEO-FFI-R) [25]. In this revised version, 14 of the 60 items were slightly adapted. Nevertheless, both inventories are highly similar and in the seminal study introducing the NEO-FFI-R [25], and in a subsequent study with Swiss and Spanish patients, results of the NEO-FFI-R the NEO-FFI were comparable [26].

### Outcome measures

We defined depressive symptoms and clinical outcomes following two earlier SIBDCS studies to allow a straightforward comparison of effects [7, 12]. Depressive symptoms were assessed with the depression subscale (HADS-D) of the Hospital Anxiety and Depression Scale (HADS) [27]. The HADS-D is a 7-item self-report questionnaire specifically designed to assess symptoms of depression in physically ill patients. Somatic symptoms and suicidal ideation were not included. Depressive symptoms are rated on a Likert scale from 0 (not at all) to 3 (most of the time) resulting in a total score between 0 and 21. Following well-established criteria, clinically significant depressive symptoms were defined as HADS-D ≥ 11, indicating probable moderate or severe depression [27]. The terms depression and depressive symptoms are used in reference to this definition henceforward.

Disease-related quality of life was assessed via the Inflammatory Bowel Disease Questionnaire (IBDQ) [28]. To dichotomize the IBDQ variable, scores below 170 were considered to indicate the low disease-related quality of life as scores of patients in remission normally range from 170 to 190 [29–31].

IBD flares were measured according to published composite endpoints [7–9, 12]. Active disease was defined as CDAI ≥ 150[32] or MTWAI ≥ 10[33], respectively. The first composite endpoint considers physician-reported flare, non-response to any administered therapy with consequent change of medication, new complication or new EIM (FNCE) and was reached upon the occurrence of any of these events after enrolment [7, 9, 12].

The second clinical composite endpoint comprises active disease, physician-reported flare, fistula, stenosis, surgery or new systemic therapy (AFFSST). It was reached upon the presence of active disease (see definition above), a physician reported flare, new fistula, new stenosis, new surgery, intake of systemic steroids and/or the start of therapy with a new TNF inhibitor or change of TNF inhibitors after enrolment [7, 8, 12].

Each composite endpoint was reached upon the first occurrence of at least one defining event after enrolment and thereby indicated a disease flare-up. For time-to-event analyses with Cox proportional hazards models, data were coded with right censoring. Coding of time-to-event data followed exactly two earlier SIBDCS studies [7, 12].

### Comparison of SIBDCS patients with population-based CoLaus¦PsyCoLaus data

NEO-FFI data from the SIBDCS and comparable data assessed with the revised NEO-FFI Inventory (NEO-FFI-R) by the CoLaus¦PsyCoLaus study were analysed for systematic differences in personality between IBD patients and the general population with a series of linear regression models (Fig. 1, Step 1). We applied 3 models to each personality domain: (i) a univariable model with cohort membership as an independent variable, (ii) a complete model corrected for potential confounders (see confounder set below) and (iii) a reduced model consisting of the variable combination that resulted in the lowest Akaike information criterion (AIC) value. Similarly, the prevalence of Type D personality (DS14 questionnaire[11]) amongst IBD patients was compared with the prevalence in the population-based control cohort.

### NEO-FFI cluster subcomponents and disease activity

To identify an association of NEO-FFI cluster subcomponents[22, 23] with disease activity (disease activity score) at enrolment, we first randomly split our data into a training and a validation set with a 60:40 ratio (Fig. 1, Step 2). Then we bootstrapped the training set (1000-fold resampling) and fit univariable linear regressions models[34] with *z* score normalized disease activity scores as dependent and each of the 13 cluster subcomponent scores as an independent variable (Fig. 1, Step 3). If necessary, cluster subcomponents were reversely coded (later indicated as reverse) so that higher scores on a cluster subcomponent always correlated with higher scores on disease activity. Mean bootstrap estimates of *p* values for the association of each cluster subcomponent with disease activity were obtained from the linear regression models and controlled for the false discovery rate (*n* = 13, Benjamini and Hochberg procedure). These false discovery rate-controlled *p*-values (FDR-*p*, controlled *α* < 0.05) were used as selection criteria for an IBD-relevant NEO-FFI risk score (see below).

### NEO-FFI risk score for IBD recurrence

We created a risk score based on the results of our bootstrapped screening approach (Fig. 1, Step 3). This score included all NEO-FFI cluster subcomponents that were significantly associated with higher disease activity at enrolment. The score was created by weighting each patient’s cluster subcomponent score values by multiplying them with the corresponding mean regression coefficients resulting from the univariable linear regression model fittings (derived from 1000 bootstrap samples) [34]. Finally, obtained values were multiplied by 10 for better readability. For each patient of the validation set the NEO-FFI risk score was calculated by summing up the weighted cluster subcomponent scores (Fig. 1, Step 4 and Supplementary Table 3). This new *NEO-*FFI risk score was validated using the validation set by implementing a univariable linear regression model with disease activity as a dependent and NEO-FFI risk score as an independent variable. For further analyses, we dichotomized the NEO-FFI risk score into two groups: low vs. high risk. Due to the underlying continuous cluster subcomponent scores, no inherent cut-off separating both groups was expected. As the NEO-FFI risk score values were normally distributed (training and validation data) we chose the mean NEO-FFI risk score of all samples (training and validation data) as a cut-off value which will be used henceforward if not indicated otherwise.

### NEO-FFI risk score performance on training vs. validation data

For further validation, we compared the performance of our NEO-FFI risk score as well as its cut-off as a classifier on training and validation data separately. We examined the score’s ability to identify patients meeting the respective endpoints within 5 years by calculating the area under the curve (AUC) and depicting corresponding receiver operating characteristic (ROC) curves (Fig. 1, Steps 5 and 6).

### Time-to-event analyses

To compare the hazards of IBD recurrence between NEO-FFI risk groups, we performed time-to-event analyses (Fig. 1, Step 6) using the Survival R package version 2.38 [35]. For this, we only used validation data (i.e. data not used for the creation of the NEO-FFI risk score). We implemented multivariable Cox proportional hazards models with different indicators of IBD recurrence as endpoints (see outcome measures). Based on clinical expertise and prior SIBDCS studies [7, 12] we chose the following control variables for multivariable models (confounder set): Type D personality status, sex, IBD diagnosis, time since IBD diagnosis, age, BMI, disease-related surgery prior to enrolment, smoking status, daily alcohol consumption. We obtained adjusted *p* values (*q* values or simply *q*) by applying the Bonferroni procedure for multiple testing, correcting for 4 clinical (*q* = *p* × 4) and 2 psychological (*q* = *p* × 2) endpoints, respectively. A sensitivity analysis of these models was performed by additionally controlling for depressive symptoms at enrolment to estimate a possible conceptual overlap of the NEO-FFI risk groups and depression.

We distinguished between occurrence and new occurrence of events. Occurrence refers to the presence of a certain variable value independent of its antecedent value, while new occurrence refers only to the presence of a certain variable value following at least one measurement with the absence of this variable value. Clinical IBD recurrence was defined by the first occurrence of active disease (CDAI ≥ 150/MTWAI ≥ 10), the first new occurrence of any EIM or the first flare according to a composite endpoint definition (see above) at follow-up. Moreover, we tested the first occurrence of depressive symptoms (HADS-D ≥ 11) and the first occurrence of low wellbeing (IBDQ < 170).

### Cumulative incidence analyses

We implemented cumulative incidence analyses (Fig. 1, Step 5) to summarize the burden of repeated exposure to indicators for deteriorating IBD course (see outcome measures) over time. For this, we adapted the statistical methods of an earlier study[36] to estimate the sum of cumulative incidence. Only validation data were used for these analyses. We bootstrapped the cumulative incidence analyses (1000-fold resampling) and calculated the median sum of cumulative incidence and its 95% confidence interval for the groups with low and high NEO-FFI risk scores over time. All calculations were performed using *R* version 4.1.0.

## Results

### Study participants

We included 1154 SIBDCS patients for whom at least 1 NEO-FFI subcomponent had been assessed. Overall data completeness of individual subcomponents was 98.3% and for 88.6% of the patients all subcomponent information was available. Depending on the outcome measure median follow-up time ranged between 5.1 and 8.2 years (for detailed survival times see Table 1). Baseline characteristics of all SIBDCS patients with available NEO-FFI risk score data (*N* = 1119) as well as patients of the SIBDCS validation subset *(N* = 456), which was used for all analyses with the newly constructed NEO-FFI risk score, are presented in Table 1. We confirmed a mixed IBD population (498 with UC/IBD-U, 621 with CD) with mild, moderate, and severe disease.

**Table 1:** Characteristics of SIBDCS study participants

Variables at enrolment (validation data) Median (1st quartile-3rd quartile); min–max		SIBDCS^**a**^ training data subset (*N* = 663)	SIBDCS validation data subset (*N* = 456)		
			Low NEO-FFI risk (*N* = 221)	High NEO-FFI risk (*N* = 235)	*P* value
NEO-FFI risk groups (Cut-off = 11.3)^**b**^	High risk	319/663 (48.1%)	–	–	–
NEO-FFI risk score	Across groups	–	11.5 (8.8–14.1); 2.7–22.1		–
		11.1 (8.8–13.4); 0.0–24.5	8.8 (7.4–9.9); 2.7–11.3	14.0 (12.5–15.9); 11.3–22.1	By definition-
Type D personality	Yes	192/654 (29.4%)	29/219 (13.2%)	113/228 (49.6%)	**< 0.001**
Diagnosis	CD UC/IBD-U	380/663 (57.3%)	116/221 (52.5%)	125/235 (53.2%)	0.995
		283/663 (42.7%)	105/221 (47.5%)	110/235 (46.8%)	
Sex	Male	327/663 (49.3%)	127/221 (57.5%)	80/235 (34.0%)	**< 0.001**
Age (years)		41.6 (31.0–52.6); 16.5–84.6	39.6 (30.4–53.0); 17.8–81.8	41.9 (31.3–51.9); 14.8–80.6	0.746
Time since diagnosis (years)		7.9 (3.0–15.9); 0.1–52.5	7.1 (2.1–15.3); 0.0–43.4	9.7 (2.8–18.3); 0.1–48.7	**0.042**
BMI (kg/m^2^)		23.1 (20.9–26.0); 14.7–49.5	23.8 (21.6–26.1); 17.3–55.1	22.8 (20.7–26.3); 15.8–44.8	0.053
Smoking status	Smoker	185/659 (28.1%)	53/219 (24.2%)	74/232 (31.9%)	0.087
Daily alcohol consumption	Yes	47/655 (7.2%)	24/217 (11.1%)	17/231 (7.4%)	0.233
Disease activity	CDAI (CD patients)	34.0 (13.0–73.3); 0.0–226.0	34.0 (16.0–78.5); 0.0–198.0	53.0 (25.0–106.0); − 0.0 to 450.0	**0.007**
	MTWAI (UC/IBD-U patients)	2.5 (1.0–5.0); 0.0–16.0	2.0 (1.0–4.5); 0.0–19.0	3.0 (2.0–6.0); 0.0–18.0	**0.049**
Complications at or prior to enrolment	Yes	217/508 (42.7%)	78/174 (44.8%)	97/184 (52.7%)	0.166
Current treatment with systemic steroids	Yes	152/505 (30.1%)	32/171 (18.7%)	48/183 (26.2%)	**0.049**
Current treatment with TNF inhibitors	Yes	85/505 (16.8%)	37/171 (21.6%)	43/183 (23.5%)	0.771
Number current therapies		1.0 (1.0–2.0); 0.0–6.0	2 (1–2); 0–5	2 (1–2); 0–7	0.076
Disease-related surgery prior to enrolment	Yes	175/508 (34.4%)	55/174 (31.6%)	67/184 (36.4%)	0.397
Fistula at or prior to enrolment	Yes	141/508 (27.8%)	45/174 (25.9%)	52/184 (28.3%)	0.700
Stenosis at or prior to enrolment	Yes	107/508 (21.1%)	34/174 (19.5%)	52/184 (28.3%)	0.071
EIM at or prior to enrolment	Yes	182/508 (35.8%)	68/174 (39.1%)	88/184 (47.8%)	0.119
Depressive symptoms (HADS-D ≥ 11)	Yes	45/655 (6.9%)	5/220 (2.3%)	30/231 (13.0%)	** < 0.001**
IBDQ score		182.0 (156.0–200.0);53.0–223.0	194.5 (171.0–206.0);87.0–222.0	169.0 (143.2–190.0);68.0–224.0	** < 0.001**
Low IBDQ (IBDQ < 170)	Yes	229/652 (35.1%)	49/220 (22.3%)	118/230 (51.3%)	** < 0.001**
Marital status	Married	327/639 (52.2%)	108/217 (49.8%)	119/225 (52.9%)	0.575
Academic education	Yes	92/646 (14.2%)	39/219 (17.8%)	26/226 (11.5%)	0.080
Physically active	Yes	411/640 (64.2%)	143/212 (67.5%)	153/224 (68.3%)	0.930
Observation time (years) for different endpoints	Active disease^**c**^	8.2 (6.1–9.6); 0.8–11.2	8.3 (6.1–9.7); 1.1–11.2	8.8 (7.2–9.8); 1.0–11.1	0.152
	Any new EIM^**c**^	8.1 (6.1–9.5); 0.8–11.2	8.2 (6.1–9.6); 1.1–11.1	8.5 (7.1–9.8); 1.0–11.1	0.115
	FNCE^**c**^	5.1 (3.0–7.2); 0.4–11.2	5.1 (2.9–7.6); 0.8–10.7	5.5 (2.8–7.8); 0.8–11.1	0.361
	AFFSST^**c**^	5.2 (3.3–7.5); 0.5–11.2	5.5 (3.1–7.6); 0.9–11.2	5.8 (3.1–7.5); 0.76–11.1	0.931
	Depressive symptoms (HADS-D ≥ 11)	5.1 (3.3–6.6); 0.6–8.1	5.4 (3.8–6.5); 0.8–9.4	5.0 (3.0–6.2); 0.7–8.0	0.143
	Low IBDQ (IBDQ < 170)	5.1 (3.2–6.6); 0.6–8.1	5.4 (3.8–6.6); 0.8–9.4	5.0 (3.0–6.2); 0.7–8.0	0.074

*P* values were calculated using the Mann–Whitney *U* or *χ*^2^ tests, respectively and refer to differences between NEO-FFI risk score groups observed within the validation data subset. When characteristics of the training and validation subset were compared, higher CDAI scores (median: training data = 34.0 vs. validation data = 45.0, *p* = 0.011), more EIM at or prior to enrolment (training data = 35.8% vs. validation data = 43.6%, *p* = 0.026) and more individuals with current anti-TNF treatment (training data = 16.8% vs. validation data = 22.6%, *p* = 0.043) in the validation data subset were observed. No other significant differences were detected. Observation numbers diverging from 1119 for the whole SIBDCS are due to missing data for certain variables

*CD* Crohn’s disease, *UC* ulcerative colitis, *IBD-U* IBD unclassified, *BMI* body mass index, *CDAI* Crohn’s disease activity index, *EIM* extraintestinal manifestation, *MTWAI* modified truelove and witts severity Index, *NEO-FFI* NEO-five-factor inventory, *TNF* tumour necrosis factor, *HADS-D* hospital anxiety and depression scale subscale for depressive symptoms

^**a**^1154 SIBDCS patients for which at least 1 NEO-FFI cluster subcomponent had been assessed were included. However, only the 1119 patients with data for all 3 cluster subcomponents (i.e., self-reproach, negative affect and activity) on which the NEO-FFI risk score is based are presented here

^**b**^The cut-off value (11.3) to interpret NEO-FFI risk groups is the mean NEO-FFI risk score of all included SIBDCS patients (training and validation data)

^**c**^Precise definitions of the respective clinical endpoint can be found in the Methods

Bold values indicate significant *P* values (i.e. *P* < 0.05)

### Personality differences between SIBDCS patients and normal population (CoLaus¦PsyCoLaus)

**Fig. 2:**
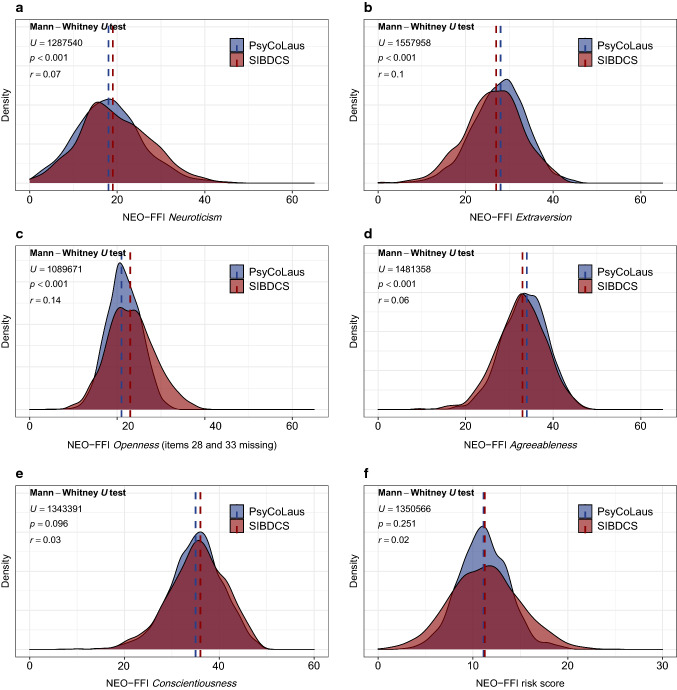
Personality domains and NEO-FFI risk scores of IBD patients and population-based controls. Density plots for the personality domains Neuroticism (**a**), Extraversion (**b**), Openness (**c**), Agreeableness (**d**) and Conscientiousness (**e**) as well as for the NEO-FFI risk score (**f**) stratified for the indicated cohorts. Red/blue dashed lines indicate median score values. Analyses: Mann–Whitney *U* test. *NEO-FFI* NEO five-factor inventory, *p* *p* value, *r* Pearson’s *r*, *SIBDCS* swiss IBD cohort study, *U* *U* statistic

Characteristics of the CoLaus¦PsyCoLaus comparison sample from the general population are presented in comparison with the SIBDCS data (training and validation data) in Table 2. Data suggest that the two cohorts significantly differed regarding most personality domains and Type D personality prevalence (SIBDCS = 30.5%, CoLaus¦PsyCoLaus = 15.4%; Fig. 2, Supplementary Fig. 1, Table 2). Further, the two cohort samples differed substantially in several socio-economic parameters such as the age structure as well as the distribution of sex, alcohol consumption or education (Table 2).

**Table 2:** Comparison of cohort characteristics

Median (1st quartile-3rd quartile); min–max		SIBDCS	CoLaus¦PsyCoLaus	Pearson’s *r* (Pr)/Cramer’s *V* (CV)	*P* value
Neuroticism SIBDCS *N* = 1119 CoLaus¦PsyCoLaus *N* = 2535		19.0 (14.0–25.0); 0–46.0	18.0 (13.0–23.0); 0–45.0	Pr = 0.074	**< 0.001**
Extraversion SIBDCS *N* = 1097 CoLaus¦PsyCoLaus *N* = 2535		27.0 (22.0–31.0); 1.0–43.0	28.0 (24.0–32.0); 3.0–45.0	Pr = 0.096	**< 0.001**
Openness^**a**^ SIBDCS *N* = 1076 CoLaus¦PsyCoLaus *N* = 2471		23.0 (19.0–27.0); 5.0–40.0	21.0 (19.0–24.0); 7.0–33.0	Pr = 0.144	**< 0.001**
Agreeableness SIBDCS *N* = 1083 CoLaus¦PsyCoLaus *N* = 2535		33.0 (29.0–36.0); 9.0–48.0	18.0 (30.0–37.0); 12.0–48.0	Pr = 0.063	**< 0.001**
Conscientiousness SIBDCS *N* = 1098 CoLaus¦PsyCoLaus *N* = 2535		36.0 (32.0–40.0); 10.0–48.0	35.0 (32.0–39.0); 7.0–48.0	Pr = 0.028	0.096
NEO-FFI(-R) risk score SIBDCS *N* = 1119 CoLaus¦PsyCoLaus *N* = 2473		11.2 (8.8–13.6); 0.0–24.5	11.1 (9.3–12.9); 2.6–22.1	Pr = 0.019	0.251
NEO-FFI(-R) risk groups (Cut-off = 11.3)^**b**^ SIBDCS *N* = 1119 CoLaus¦PsyCoLaus *N* = 2473	High risk	554/1119 (49.5%)	1148/2473 (46.4%)	CV = 0.029	0.093
Type D personality SIBDCS *N* = 1101 CoLaus¦PsyCoLaus *N* = 2578	Yes	336/1101 (30.5%)	397/2578 (15.4%)	CV = 0.173	** < 0.001**
Sex SIBDCS *N* = 1119 CoLaus¦PsyCoLaus *N* = 2600	Male	534/1119 (47.7%)	1111/2600 (42.7%)	CV = 0.046	**0.006**
Age (years) SIBDCS: *N* = 1119 CoLaus¦PsyCoLaus: *N* = 2600		41.3 (31.0–52.6); 14.8–84.6	59.4 (41.5–67.6); 41.5–54.2	**Pr = 0.515**	**< 0.001**
BMI (kg/m^2^) SIBDCS *N* = 1081 CoLaus¦PsyCoLaus *N* = 2544		23.2 (21.0–26.1); 14.7–55.1	25.4 (22.8–28.3); 15.8–44.8	Pr = 0.217	**< 0.001**
Smoking status SIBDCS *N* = 1110, CoLaus¦PsyCoLaus *N* = 2558	Smoker	312/1110 (28.1%)	507/2558 (19.8%)	CV = 0.091	**< 0.001**
Daily alcohol consumption SIBDCS: *N* = 1103, CoLaus¦PsyCoLaus: *N* = 2573	Yes	88/1103 (8.0%)	726/2573 (28.2%)	CV = 0.223	**< 0.001**
Academic education SIBDCS *N* = 1091, CoLaus¦PsyCoLaus *N* = 2599	Yes	157/1091 (14.4%)	706/2599 (27.2%)	CV = 0.138	**< 0.001**

Cohort characteristics of study participants from the SIBDCS at enrolment and the CoLaus¦PsyCoLaus cohort at NEO-FFI assessment. *P* values were calculated using the Mann–Whitney *U* or *χ*^2^ tests, respectively. Effect sizes were calculated as Pearson’s *r* or Cramer’s *V*, respectively. Numbers diverging from 1154 (SIBDCS) or 2600 (CoLaus¦PsyCoLaus), respectively, are due to missing data for certain variables

*NEO-FFI* NEO-five-factor inventory, *SIBDCS* swiss IBD cohort study

^**a**^NEO-FFI items 28 and 33 are missing because they were not assessed in the SIBDCS (compare limitations)

^**b**^The cut-off value (11.3) to interpret NEO-FFI risk groups is the mean NEO-FFI risk score of all included SIBDCS patients (training and validation data)

Bold values indicate significant *P*-values (i.e. *P* < 0.05) or moderate-high correlations (i.e. Pr/CV > 0.500), respectively

To further understand observed differences in personality data between SIBDCS patients and the general population (Table 2), we applied a series of linear multivariable regression models adjusted for potential confounders (see Methods). In these models, significant differences between both cohorts remained with lower scores for Extraversion, higher scores for the partially reconstructed (items 28 and 33 missing) Openness as well as lower scores for the Agreeableness domain in SIBDCS patients (Supplementary Table 4). Type D personality status was significantly more frequent in SIBDCS patients [multivariable model: odds ratio (OR) = 1.86, CI: 1.49–2.32, *p* < 0.001, Supplementary Table 5].

Given that CoLaus¦PsyCoLaus data were exclusively acquired in Lausanne, we directly compared the SIBDCS subsample from Lausanne to CoLaus¦PsyCoLaus data. In this comparison, higher scores of SIBDCS patients in the Openness domain were also observed (*p* < 0.001); however, other comparisons were not statistically different, most likely due to low SIBDCS subsample sizes (*N* = 61–64, Supplementary Fig. 2). Type D personality was more frequent amongst patients with IBD also in the Lausanne subsample (Supplementary Fig. 1) confirming the results for the whole IBD dataset.

### NEO-FFI cluster subcomponent screening and construction of the NEO-FFI risk score

To identify which of the 13 personality traits were associated with high disease activity (standardized CDAI/MTWAI score at enrolment), we applied a bootstrap screening approach to the SIBDCS training set (see Methods). Three subcomponents were identified, namely activity (reverse, regression coefficient (RC) = 0.055, *R*^2^ = 0.035, FDR-*p* = 0.023), self-reproach (RC = 0.030, *R*^2^ = 0.032, FDR-*p* = 0.023) and negative affect (RC = 0.043, *R*^2^ = 0.023, FDR-*p* = 0.023), Supplementary Fig. 3, panels a–c). These three cluster subcomponents were then combined to a NEO-FFI risk score (Supplementary Table 3), which was associated with higher disease activity levels at enrolment when applied to the validation data set (*R*^2^ = 0.050, *p* = 0.004, Supplementary Fig. 3, panel d).

Patient data were stratified according to a patient’s NEO-FFI risk group status, using the mean score of all SIBDCS patients (11.3 points) as a cut-off. NEO-FFI high-risk status was significantly associated with Type D personality, sex (female), disease activity, depressive symptoms, and lower disease-related quality of life (Table 1). There was no difference between CD and UC/IBD-U patients regarding the NEO-FFI risk score. NEO-FFI high-risk status was associated with longer disease duration but there were no associations of the NEO-FFI risk score with indicators of more severe prior disease courses such as prior abdominal surgery, fistula or stenosis, prior complications or prior EIMs (Table 1). Moreover, NEO-FFI risk score values did not differ between IBD patients and CoLaus¦PsyCoLaus participants (median: SIBDCS: 11.2, CoLaus¦PsyCoLaus: 11.1, *p* = 0.251, Table 2).

We applied the NEO-FFI risk score categorisation as a predictor of outcome measures. As expected, for SIBDCS patients without risk stratification pronounced intraindividual fluctuation of CDAI, MTWAI, HADS-D and IBDQ were apparent but no systematic increase or decrease of values over time could be observed (descriptive). When applying the NEO-FFI risk score categorisation, however, there were stable trends showing that patients at high NEO-FFI risk on average suffered from higher disease activity (CDAI, MTWAI), higher depression scores (HADS) as well as the lower disease-related quality of life (IBDQ, descriptive, Supplementary Figs. 4, 5). We calculated receiver operating characteristic curves and observed strong predictive power for both future depressive symptoms (AUC = 0.77–0.87) and episodes of low disease-related quality of life (AUC 0.74–0.75) but only modest power for active disease, new EIMs or composite endpoints (FNCE or AAFST, AUC = 0.55–0.68, Supplementary Fig. 6).

### NEO-FFI high-risk group with higher hazards for IBD recurrence

**Fig. 3:**
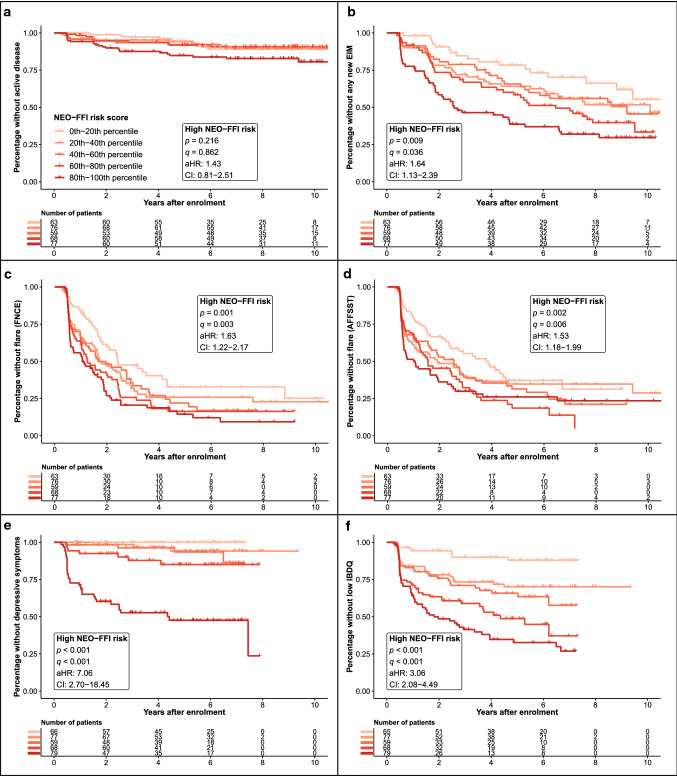
High NEO-FFI risk increases the hazards for clinical deterioration. Kaplan Meier curves corrected for confounders including Type D personality (see methods) for active disease (**a**), new EIMs (**b**) FNCE flares (**c**), AFFSST flares (**d**), depressive symptoms (**e**) and low IBDQ (**f**) are presented. For the graphics, IBD patients are stratified into 5 groups according to NEO-FFI risk score 20 percentile intervals. The estimates describe the comparison of the high NEO-FFI risk group with the low NEO-FFI risk group (cut-off 11.3). Depressive symptoms were defined as HADS-D ≥ 11. Low IBDQ was defined as values below 170. Analyses: multivariable Cox proportional hazards models. *AFFSST* active disease, physician-reported flare, new fistula, new stenosis, surgery, or new systemic therapy, *aHR* adjusted hazard ratio, *CDAI* Crohn’s disease activity index, *CI* confidence interval, *EIM* extraintestinal manifestation, *FNCE* physician reported flare, non-response to therapy, *HADS* hospital anxiety and depression scale, *IBD* inflammatory bowel disease, *IBDQ* inflammatory bowel disease questionnaire, *MTWAI* modified truelove and witts severity index, *NEO-FFI* NEO five-factor inventory

In univariable time-to-event analyses, patients belonging to the NEO-FFI high-risk group shared higher hazard ratios for all endpoints tested (*q* ≤ 0.011, not shown). In fully corrected Cox proportional hazards models (including correction for Type D personality) analysing the same endpoints, higher NEO-FFI risk scores remained significantly associated with the new occurrence of any new EIM (adjusted hazard ratio (aHR) = 1.64, *q* = 0.036, Fig. 3, panel b) but not with active disease (CDAI ≥ 150/MTWAI ≥ 10, aHR = 1.43, *q* = 0.862, Fig. 3, panel a). High NEO-FFI risk was also associated with our composite endpoints FNCE (aHR = 1.63, *q* = 0.003, Fig. 3, panel c) and AFFSST (aHR = 1.53, *q* = 0.006, Fig. 3, panel d). Finally, high NEO-FFI risk predicted depressive symptoms (HADS ≥ 11, aHR = 7.06, *q* < 0.001, Fig. 3, panel e) and low disease-related quality of life (IBDQ < 170, aHR = 3.06, *q* < 0.001, Fig. 3, panel f).

We performed sensitivity analyses to assess the NEO-FFI high-risk’s predictive power in patients who were in remission (CDAI < 150/ MTWAI < 10) at enrolment (Supplementary Fig. 7, panels a–f). Significant effects remained robust for the two composite endpoints FNCE and AFFSST (Supplementary Fig. 7, panels c, d) as well as for new depressive symptoms and low disease-related quality of life (Supplementary Fig. 7, panels e, f) for this subgroup of patients. Effects regarding any new* EIM* remained significant and were only lost after correction for multiple testing (*p* = 0.022, *q* = 0.087, Supplementary Fig. 7, panels b).

In further sensitivity analyses, we controlled for depressive symptoms and replaced the variable Type D personality with depressive symptoms at enrolment (HADS ≥ 11) in our multivariable Cox proportional hazards models. The observed associations of a higher NEO-FFI risk score with higher hazards for outcomes remained robust except for the endpoint of any new EIM where significance was lost after adjusting for multiple testing (*p* = 0.028, *q* = 0.112, data not shown).

Furthermore, we performed post hoc analyses to investigate the predictive power of the NEO-FFI risk score for individual EIMs (Supplementary Fig. 8). For most EIMs we observed an overall trend that a higher NEO-FFI risk score was associated with higher hazards; however, significance was only observed for arthritis/arthralgia as the most frequent EIMs.

### NEO-FFI high-risk group with a higher cumulative disease burden

**Fig. 4:**
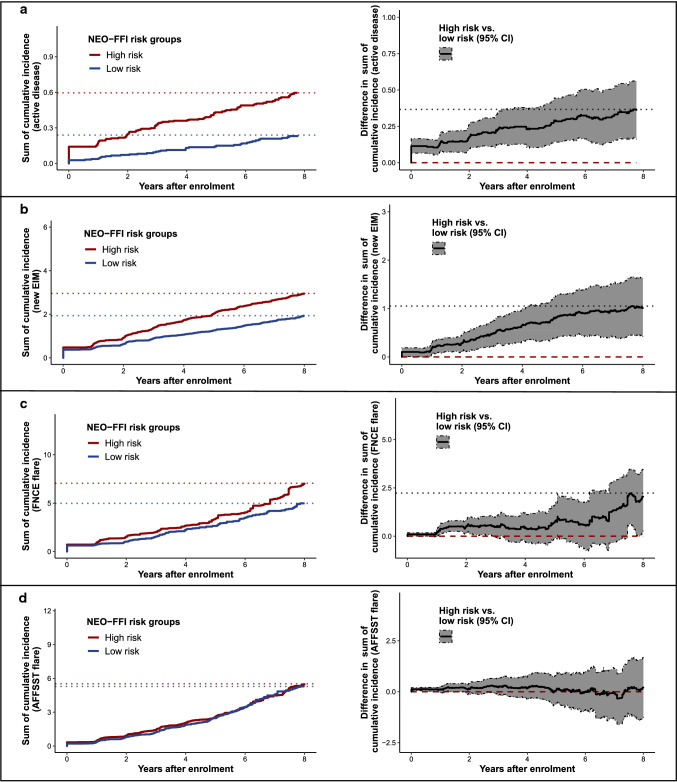
High NEO-FFI risk increases the cumulative burden of disease. Plots illustrating the cumulative incidence counts (left) and the differences of cumulative incidence for different endpoints between low and high NEO-FFI risk groups (right) for active disease (**a**), new EIMs (**b**) FNCE flares (**c**) and AFFSST flares (**d**) in IBD patients. Dotted lines in the left plots indicate the maximal values observed and the red dashed line in the right plots mark the zero-difference line. Overlap of the zero-difference line and the 95% confidence interval indicates a lack of significance. Results were obtained by bootstrapping with 1000-fold resampling. Analyses: cumulative incidence analyses. *AFFSST* active disease, physician-reported flare, new fistula, new stenosis, surgery or new systemic therapy, *CDAI* Crohn’s disease activity index, *CI* confidence interval, *EIM* extraintestinal manifestation, *FNCE* physician reported flare, non-response to therapy, new complication or EIM, *IBD* inflammatory bowel disease, *MTWAI* modified truelove and witts severity index, *NEO-FFI* NEO five-factor inventory

**Fig. 5:**
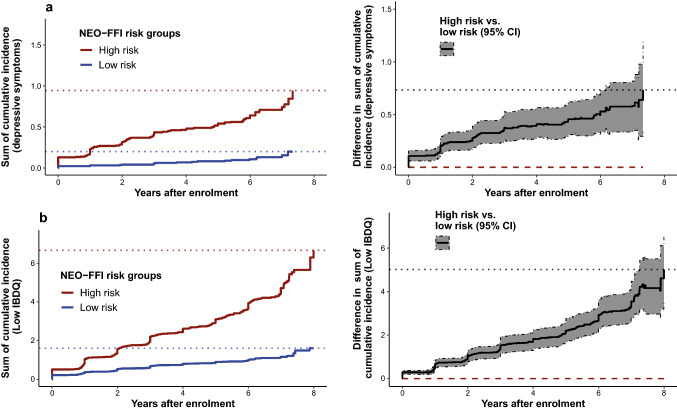
High NEO-FFI risk increases the cumulative psychological burden of disease. Plots illustrating the cumulative incidence counts (left) and the differences in cumulative incidence between low and high NEO-FFI risk groups (right) for depressive symptoms (**a**) and low IBDQ (**b**) in IBD patients. Dotted lines in the left plots indicate the maximal values observed and the red dashed line in the right plots mark the zero-difference line. Overlap of the zero-difference line and the 95% confidence interval indicates a lack of significance. Results were obtained by bootstrapping with 1000-fold resampling. Depressive symptoms were defined as HADS-D ≥ 11. Low IBDQ was defined as values below 170. Analyses: cumulative incidence analyses. *CI* confidence interval, *HADS* hospital anxiety and depression scale, *IBD* inflammatory bowel disease, *IBDQ* inflammatory bowel disease questionnaire, *NEO-FFI* NEO five-factor inventory

To assess the NEO-FFI risk score as a predictor for the cumulative burden of disease over time, we implemented a cumulative incidence analysis. We stratified patients according to the NEO-FFI risk groups and assessed disease burden by counting all recorded events of a respective endpoint over the course of up to 8 years for each patient (Figs. 4, 5).

After 7–8 years, an average patient with NEO-FFI high-risk status experienced more than 0.60 (CI 0.43–0.79) cumulative incidences of active disease, 2.96 (CI 2.50–3.46) EIMs (including new EIMs and re-occurrences of the same EIM after event-free recording), 7.06 (CI 6.17–7.94) FNCE and 5.52 (CI 4.54–6.68) AFFSST flares. Further, an average patient with NEO-FFI high-risk status experienced more than 0.94 (0.60–1.39) episodes with depressive symptoms and more than 6.66 (5.23–8.04) episodes of low disease-related quality of life (Figs. 4, 5) within 7–8 years.

Patients of the validation set belonging to the high-risk group experienced more events for most endpoints: When compared with patients with a low NEO-FFI risk score, patients with high NEO-FFI experienced 0.36 (CI 0.16–0.56) more events of active disease, 1.02 (CI 0.42–1.64) more new EIMs, more FNCE, 0.73 (0.33–1.21) more episodes with depressive symptoms and 5.02 (3.45–6.66) more episodes of low disease-related quality of life (Figs. 4, 5). The composite flare endpoint FNCE was more frequently reached in patients with high NEO-FFI risk score (difference 2.08, CI 0.27–3.51), even though the 95% confidence interval overlapped the zero-difference line at times, whereas no differences regarding the AFFSST flare endpoint were observed (difference 0.23, CI − 1.25 to 1.74, Figs. 4, 5).

In a further post hoc analysis, we investigated the exploratory endpoint of any new surgery (part of AFFSST). As expected, a trend for higher cumulative numbers of surgeries over time in patients at high NEO-FFI risk was evident; however, these differences were not significant, possibly due to the low number of surgeries per patient (Supplementary Fig. 9).

## Discussion

In our study, we identified personality subcomponents that were significantly associated with higher disease activity and could be meaningfully combined to a new NEO-FFI IBD risk score. This score showed significant predictive power for future IBD recurrence, depressive symptoms and low disease-related quality of life and a higher cumulative burden of disease during the following 7–8 years.

### The NEO-FFI risk score

We combined three out of 13 NEO-FFI cluster subcomponents (self-reproach, negative affect and low activity) to a new NEO-FFI IBD risk score, which besides Type D personality [12], is the second descriptor of personality associated with IBD activity. Type D personality comprises social inhibition and negative affectivity and thus might seem conceptually related; however, both personality descriptors were independent since the predictive power of the NEO-FFI IBD risk score remained intact even after statistical controlling for Type D personality. Further, the predictive power of both, a high NEO-FFI risk score and Type D personality regarding IBD disease course remained even after correction for depressive mood for most endpoints [12]. This indicates that the NEO-FF risk score, Type D personality and depressive symptoms each have independent predictive value regarding future IBD activity.

### Personality and quality of life

In our study, patients with high NEO-FFI risk showed higher hazards (aHR > 3.06) for experiencing episodes of low quality of life (IBDQ) and cumulated more such episodes over time. IBD can have a prolonged impact on daily functioning and earlier studies have reported lower quality of life amongst IBD patients when compared to healthy controls [37]. Further, low quality of life has been shown to be a marker of clinical[38, 39] and mucosal disease activity [39]. Thus, the association between personality and disease-related quality of life is highly relevant for IBD patients [38].

### Personality and IBD course

Similar to Type D personality [11], high NEO-FFI risk might indicate a vulnerability to stress. This seems plausible for each of the three NEO-FFI risk subcomponents: low activity might lead to reduced coping behaviour and/or less physical activity which both are disadvantageous in the context of stress [40]. Similarly, high scores in self-reproach and negative affect might increase stress vulnerability and thereby be especially relevant for IBD patients who frequently anticipate social stigmatisation. For instance, fear relating to bowel accidents is among the most commonly reported emotions in IBD patients [41]. Furthermore, low activity, self-reproach and negative affect have been correlated with low life satisfaction and loneliness [23]. A large body of evidence indicates interactions of personality, stress, depressive symptoms, inflammation and IBD recurrence[7, 8, 12, 42] and individuals with Type D personality or high NEO-FFI risk scores would be vulnerable for disruptions of these multidirectional interactions [42].

An association of personality traits and microbiota markers would be a fascinating explanation since alterations in intestinal microbiota were found to be associated with psychological impairments in SIBDCS patients [43]. These analyses are the subject of ongoing studies.

### Personality findings of IBD patients compared to the findings of a general population sample

In this study, we identified differences in three FFM personality domains between IBD patients and the population-based sample, namely lower Extraversion, higher Openness and lower Agreeableness amongst IBD patients. Moreover, Type D personality was almost two-fold more prevalent amongst IBD patients. However, one should interpret these findings with caution: (i) IBD patients were assessed after enrolment into the SIBDCS after a history of ≥ 3 months of IBD and some differences such as lower Extraversion might be an indirect consequence of some IBD symptoms (e.g., diarrhoea or abdominal pain). (iii) The observed differences in NEO-FFI domains are subtle with very weak effect sizes (i.e. Pearson's r mostly below 0.1) and low *p* values are mainly due to the high power of our study with overall 3754 participants. (iv) We observed considerable differences in Type D prevalence (30% in SIBDCS vs. 15% CoLaus¦PsyCoLaus); however, Type D prevalence was much higher in other general population samples (e.g., 20–40%) [44], highly similar compared to SIBDCS patients. (v) SIBDCS recruits Swiss IBD patients from whole Switzerland whereas all CoLaus¦PsyCoLaus participants were initially recruited in Lausanne, the second-largest city in the French-speaking part of Switzerland. Even though differences in Type D, Openness, Extraversion and Agreeableness remained robust after adjustment for confounders, a direct comparison between Lausanne SIBDCS and CoLaus¦PsyCoLaus participants was underpowered for all NEO-FFI-related outcomes.

All in all, our data can neither confirm nor exclude an IBD personality, but clinical experience shows that an IBD diagnosis cannot be suspected on the basis of a patient’s personality. Contrary to earlier beliefs (1950) when UC was regarded as one of the “holy seven” psychosomatic disorders [17], today IBD is understood as a primarily immunologically mediated disease. Nevertheless, our evidence clearly indicates that personality can substantially shape the clinical course after the first manifestation of IBD.

There was no evidence for personality differences between CD and UC/IBD-U. This represents an interesting finding in line with earlier SIBDCS findings showing similar rates of Type D personality in both IBD subtypes [12].

### Personality traits and chronic diseases

Personality traits including the NEO-FFI specifically have been demonstrated to be temporarily stable with only rare substantial changes in long-term observational studies [45]. Nevertheless, changes in personality occur and might be influenced by critical life events to some extent [45]. Since exposure to chronic diseases such as IBD constitutes life events, these mechanisms might partially explain our findings as an example of reversed causality. Indeed a study analysing pooled data of 17,000 patients showed that some chronic illnesses can lead to a change in certain personality domains after disease onset [46]. However, the underlying diseases investigated were very heterogeneous and did not include IBD. The greatest effects were observed in stroke patients where neuronal damage might partially explain the effects. Surprisingly, cancer patients did not show any significant changes in personality domains. Therefore, this study does not convey a generalizable effect of chronic illness on personality and cannot easily be transferred to IBD.

### Clinical application of the NEO-FFI risk score

Patients with a high NEO-FFI risk score are at increased risk for clinical deterioration, future depressive symptoms, and low IBD-related quality of life. These patients might benefit from closer clinical observation and regular (formal or informal) assessment regarding psychological symptoms and quality of life. In these patients, a strong patient-physician relationship might be especially relevant for good overall outcomes. Although high-quality evidence remains scarce, recent findings suggest that psychotherapy can improve relevant psychological outcomes in IBD including disease-related quality of life [47].

In any case, the NEO-FFI risk score represents a non-invasive and inexpensive marker for several adverse outcomes. Further, NEO-FFI or Type D needs to be assessed only once for each patient and could easily be applied in clinical practice for patients’ benefits.

### Strength and limitations

Our study has several strengths and limitations. A particular strength is the large sample size (3754 patients) and excellent socio-demographic and clinical characterization of both cohorts. The composite flare endpoints (FNCE, AFFSST) comprise several highly relevant clinical aspects of IBD (e.g., primary non-response to therapy, new stenosis). Further, the longitudinal assessment of clinical data over a long follow-up period enabled us to combine cross-sectional, time-to-event, and cumulative incidence analyses which increases the validity of our findings. However, limitations include methodological differences between the two cohorts (SIBDCS, CoLaus¦PsyCoLaus), as for example geographic areas for recruitment. Furthermore, there were 2 of 12 items missing for the post-factum construction of the Openness domain. Although we took this into consideration when comparing data with the CoLaus¦PsyCoLaus cohort by using the same items, this procedure will limit comparison with other studies. Moreover, even though the training and validation sets of patients with NEO-FFI risk data were strictly separated, de novo construction of our NEO-FFI risk score for IBD is a limitation and independent confirmation is required. Our results are also limited by the dichotomisation of the continuous NEO-FFI risk score for analytic reasons. Furthermore, the reverse causality of our findings (i.e., a severe disease course changes a patient’s personality) cannot be excluded. Finally, objective measures of disease activity including calprotectin, endoscopic and histologic data were not sufficiently available for SIBDCS patients but would be desirable for further studies. Therefore, it should be kept in mind that all outcomes which were associated with the NEO-FFI are at least partially subjective including the established IBD activity scores CDAI and MTWAI.

## Conclusion

In conclusion, the NEO-FFI personality subcomponents negative effect, self-reproach and low activity can predict relevant IBD outcome measures such as risk and cumulative incidence of IBD disease activity, depressive symptoms and low disease-related quality of life in IBD. Personality thus represents an easily accessible, temporally stable and inexpensive parameter that seems well suited as a clinical risk assessment tool in IBD.

## Supplementary Information

Below is the link to the electronic supplementary material.

Supplementary file1 (PDF 45 KB)

Supplementary file2 (PDF 232 KB)

Supplementary file3 (PDF 4105 KB)

Supplementary file4 (PDF 210 KB)

Supplementary file5 (PDF 262 KB)

Supplementary file6 (PDF 1591 KB)

Supplementary file7 (PDF 916 KB)

Supplementary file8 (PDF 21 KB)

Supplementary file9 (PDF 103 KB)

Supplementary file10 including all supplementary materials (DOCX 1852 KB)

## Data Availability

The data underlying this article cannot be shared publicly due to the privacy protection of individuals that participated in the study in compliance with Swiss law. The data will be shared on reasonable request to the corresponding author and the Swiss IBD cohort study or CoLaus¦PsyCoLaus study scientific committee, respectively.

## Abbreviations

AFFSST: Composite endpoint: IBD flare defined by active disease, physician-reported flare, fistula, stenosis, surgery or new systemic therapy
aHR: Adjusted hazard ratio
AIC: Akaike information criterion
AUC: Area under the curve
BMI: Body mass index
CD: Crohn’s disease
CDAI: Crohn’s disease activity index
CI: Confidence interval
e.g.: Exempli gratia (for example)
EIM: Extraintestinal manifestations
FDR: False discovery rate
FFM: Five-factor model
FNCE: Composite endpoint: IBD flare defined by physician reported flare, non-response to any administered therapy with consequent change of medication, new complication or new EIM
HADS: Hospital anxiety and depression scale
HADS-D: Depression subscale of the hospital anxiety and depression scale
aHR: Adjusted hazard ratio
HR: Hazard ratio
IBD: Inflammatory bowel disease
IBDQ: Inflammatory bowel disease questionnaire
IBD-U: IBD unclassified
i.e.: Id est (that is)
MTWAI: Modified truelove and witts severity index
NEO-FFI: NEO five-factor inventory
NEO-FFI-R: Revised NEO five-factor inventory
OR: Odds ratio
*p*: *p* Value
PSC: Primary sclerosing cholangitis
CoLaus¦PsyCoLaus: The CoLaus¦PsyCoLaus study is a cohort study that has been recruited from the general population of Lausanne
*q*: Multiple testing corrected *p* value
*R*^2^: Square of the correlation coefficient, also known as coefficient of determination
ROC: Receiver operating characteristic
*S*: Standard deviation of the sample
SIBDCS: Swiss IBD cohort study
*t*_n_: Follow-up time *n* of recording
TNF: Tumour necrosis factor
*U*: *U* Statistic
UC: Ulcerative colitis
*z*: *z* Score
*x*: A measurement
$\overline{x }$: Mean of the sample
