# Supplementary figures and images for: The personality traits activity, self-reproach, and negative affect jointly predict clinical recurrence, depressive symptoms, and low quality of life in inflammatory bowel disease patients
Source: J Gastroenterol. 2022 Jul 28;57(11):848–66. doi: 10.1007/s00535-022-01902-7 (PMC9596530; doi:10.1007/s00535-022-01902-7)

Prevalence of Type D personality

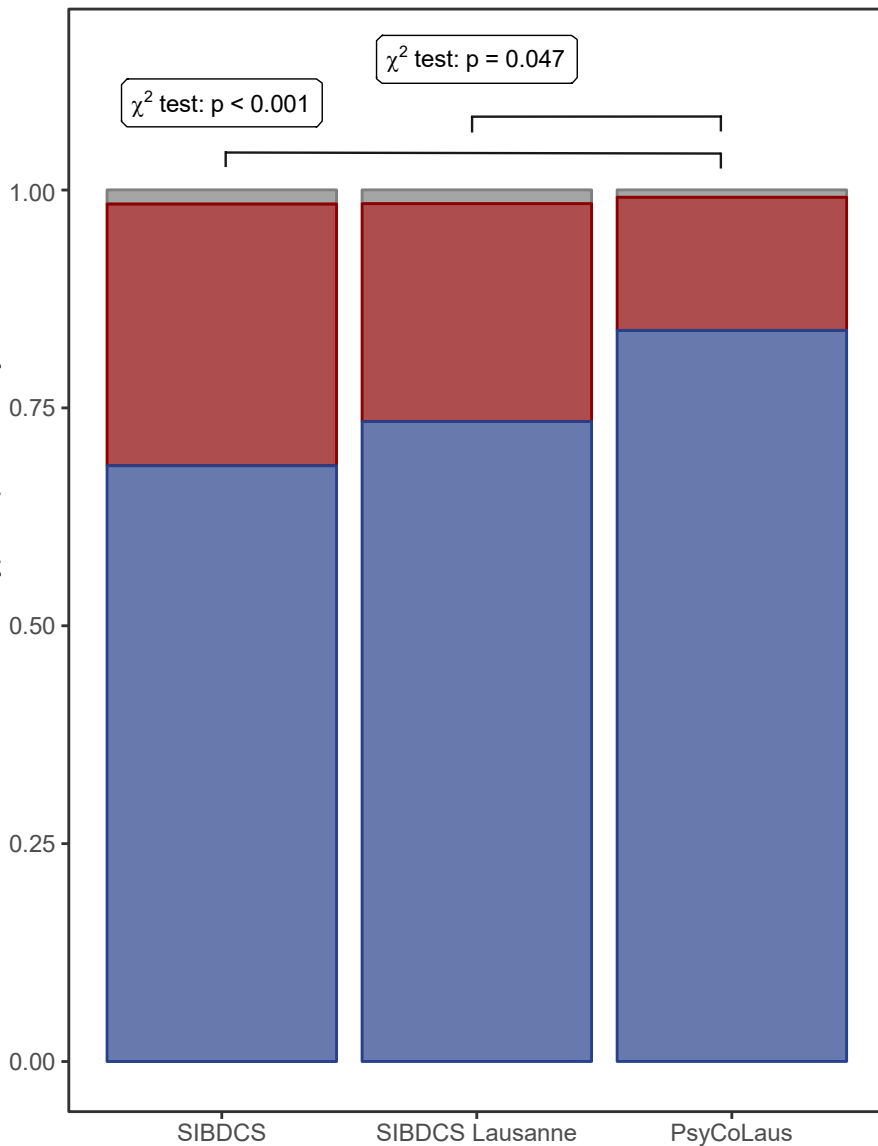

Type D personality:

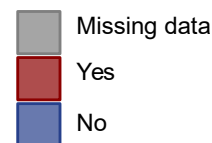

Supplement: Supplementary file 1 — Supplementary file1 (PDF 45 KB) [file 535_2022_1902_MOESM1_ESM.pdf]

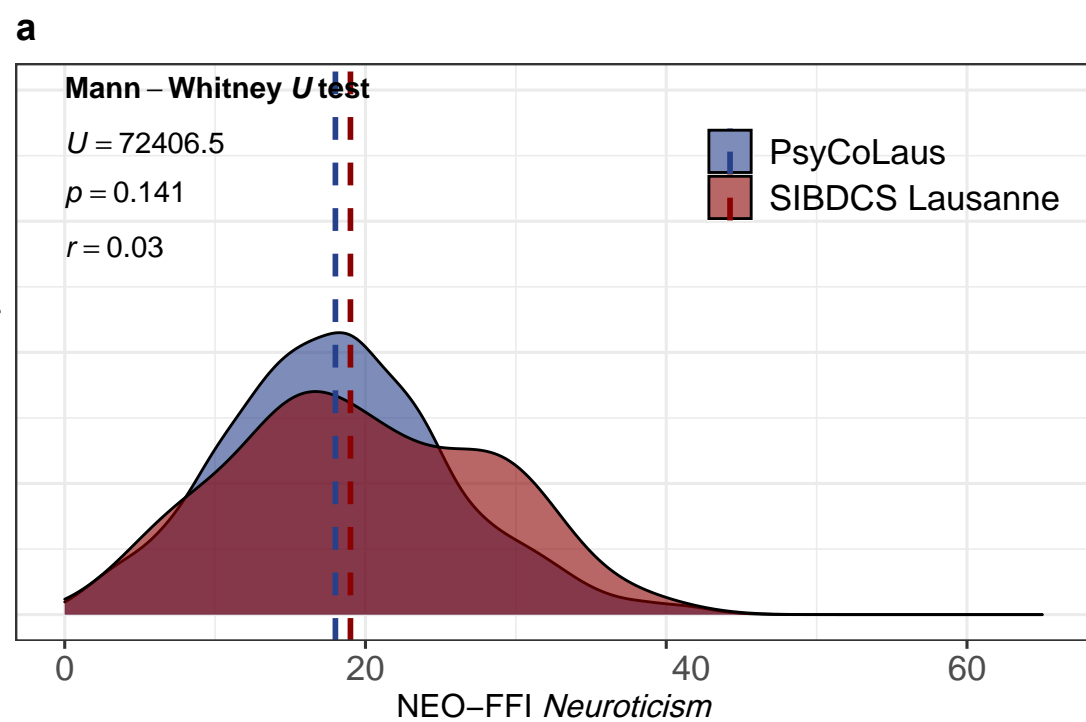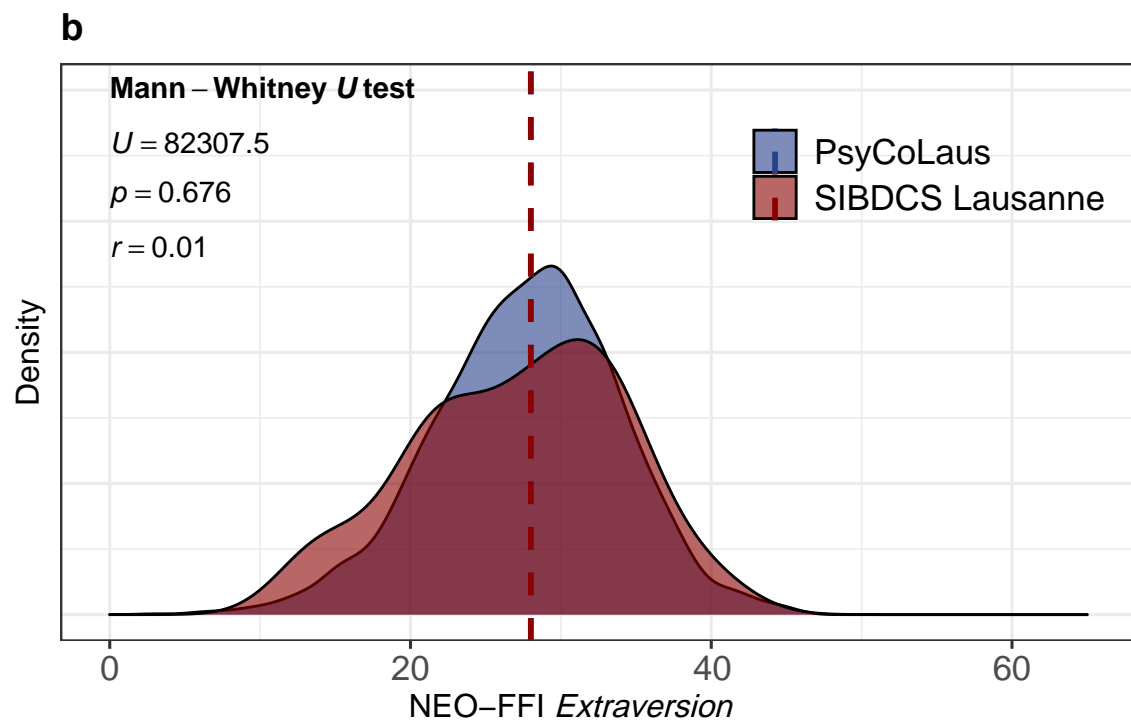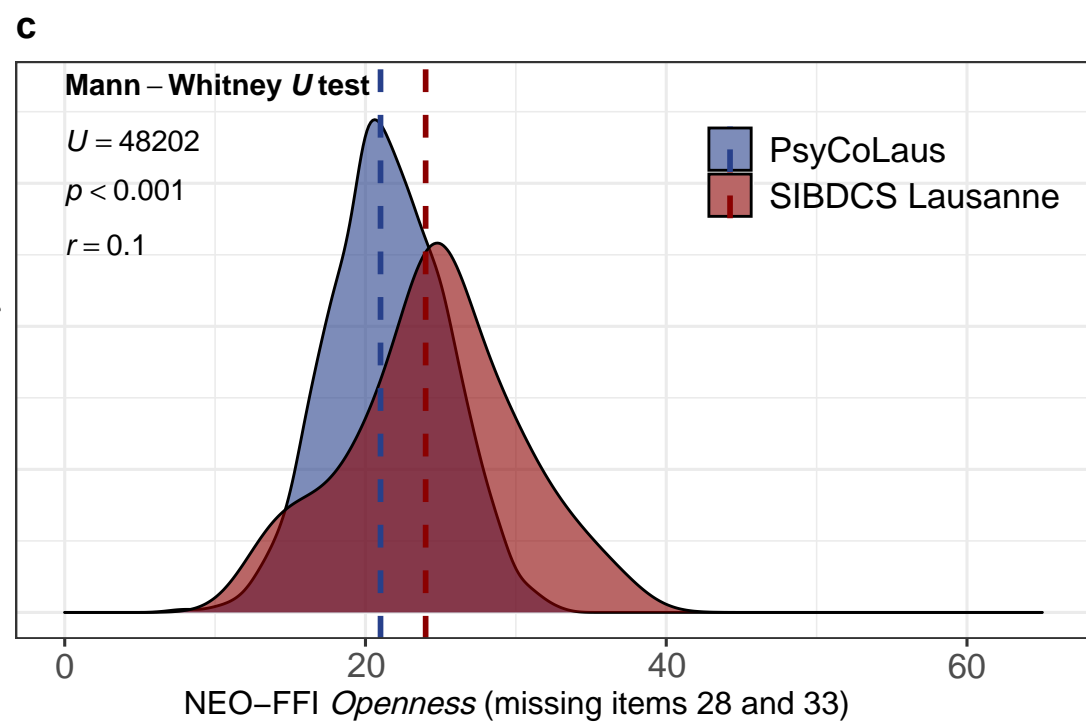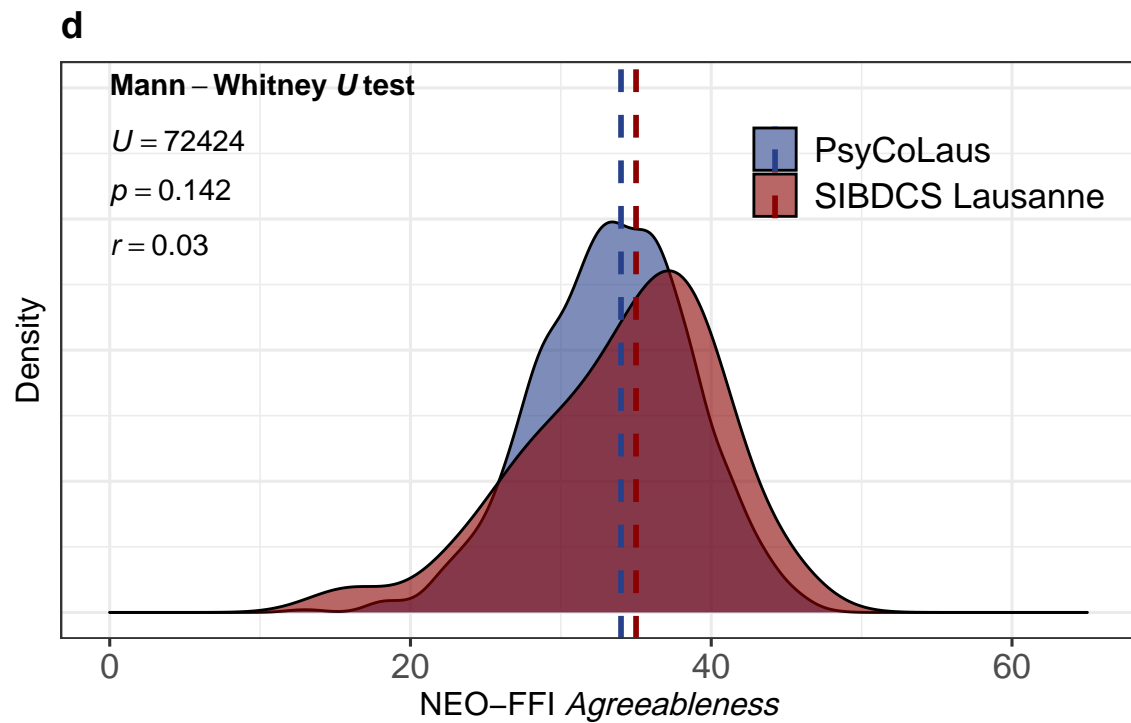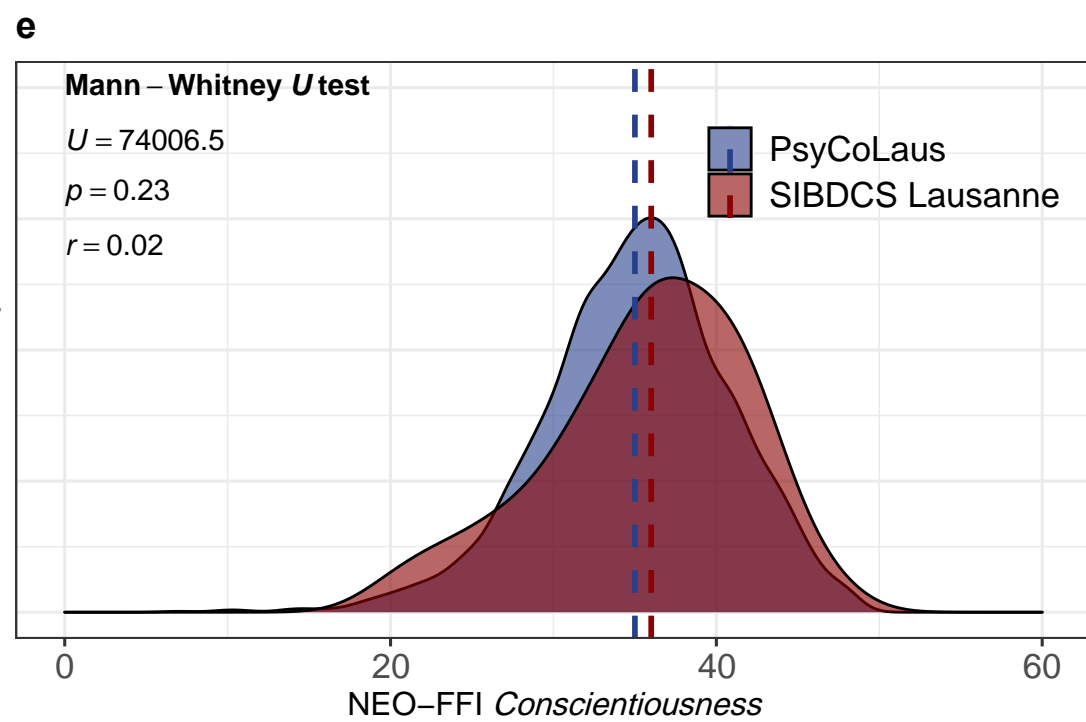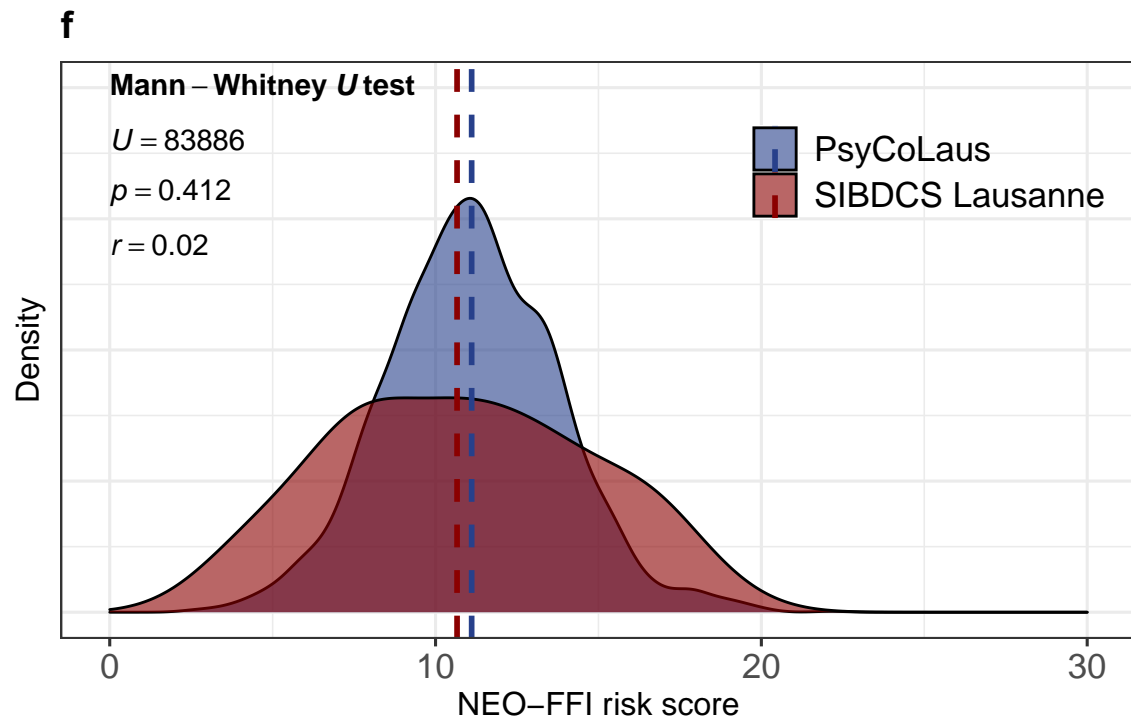

Supplement: Supplementary file 2 — Supplementary file2 (PDF 232 KB) [file 535_2022_1902_MOESM2_ESM.pdf]

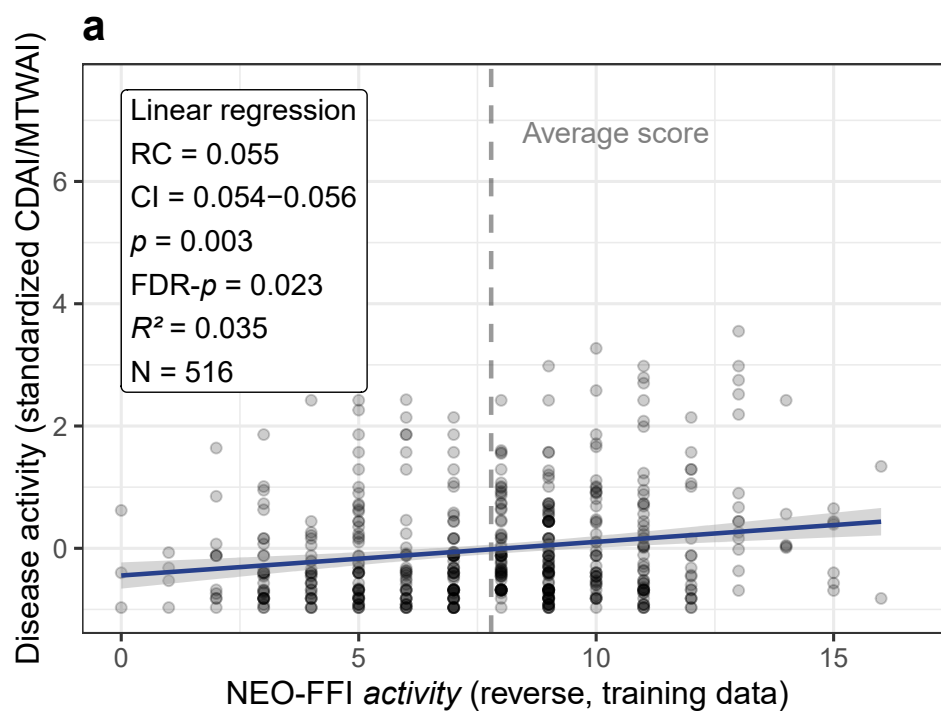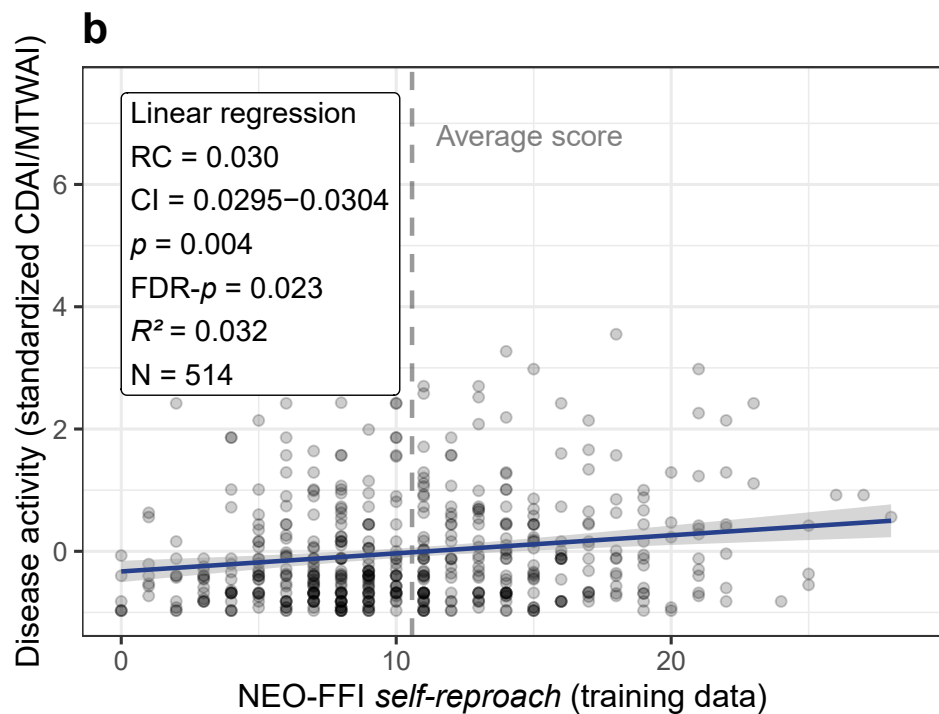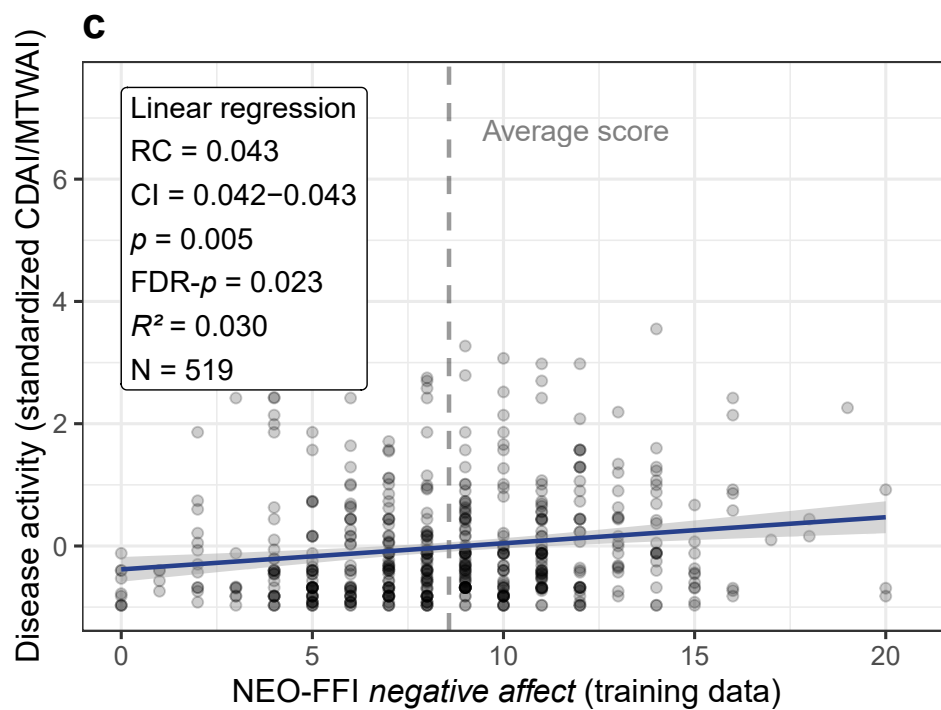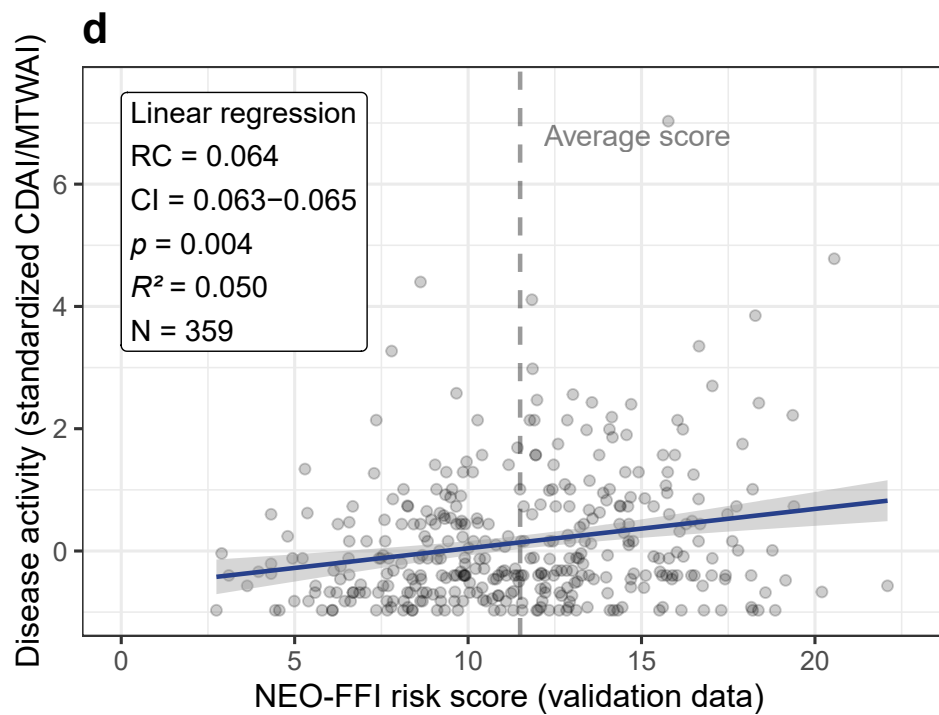

Supplement: Supplementary file 3 — Supplementary file3 (PDF 4105 KB) [file 535_2022_1902_MOESM3_ESM.pdf]

**a**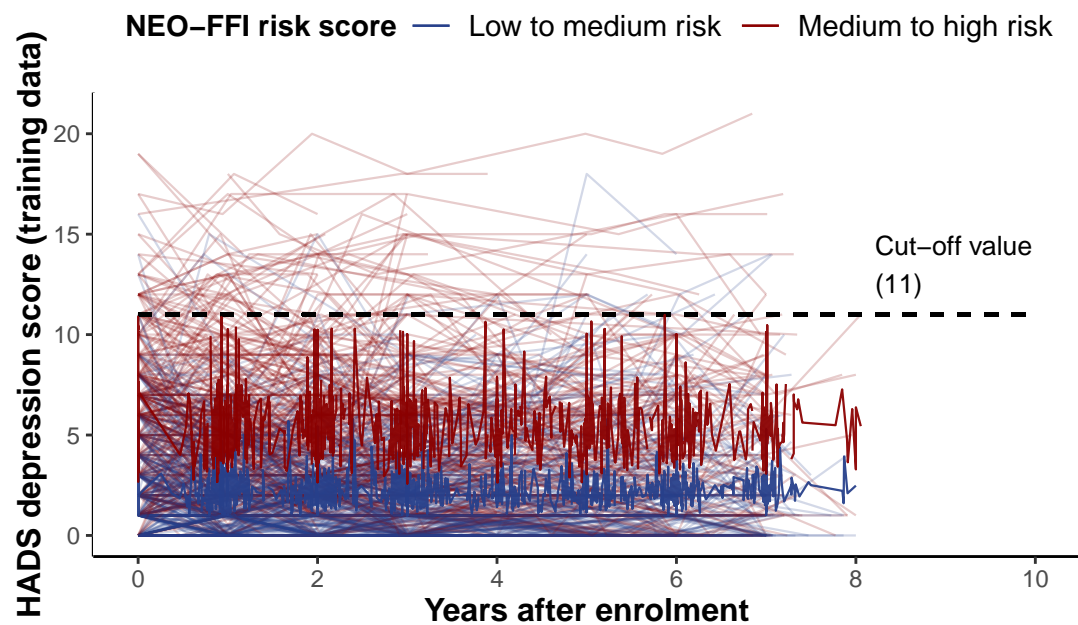**b**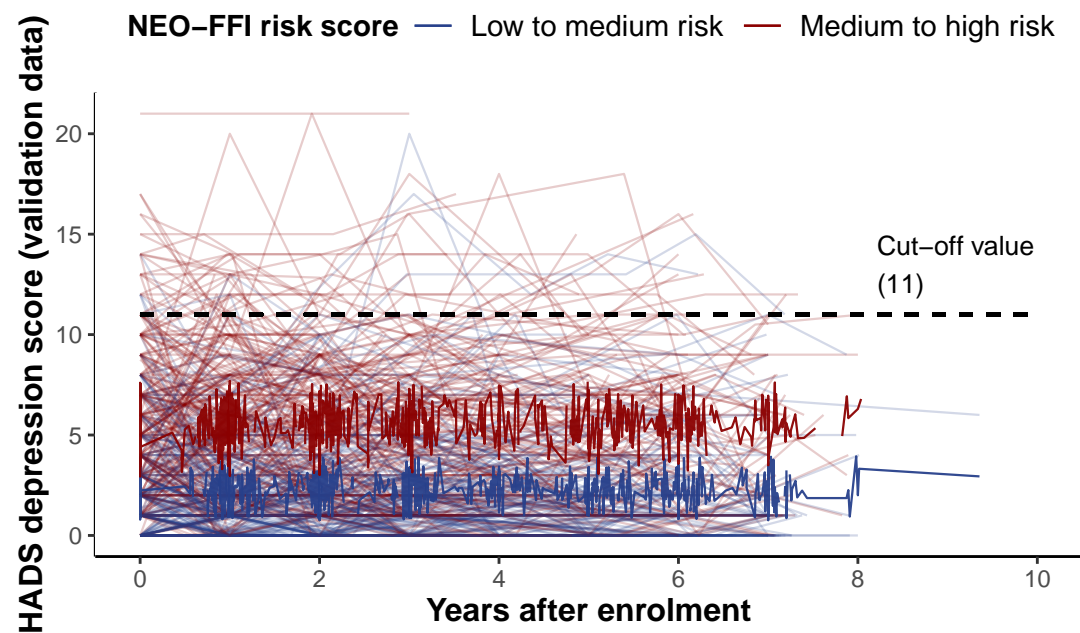**c**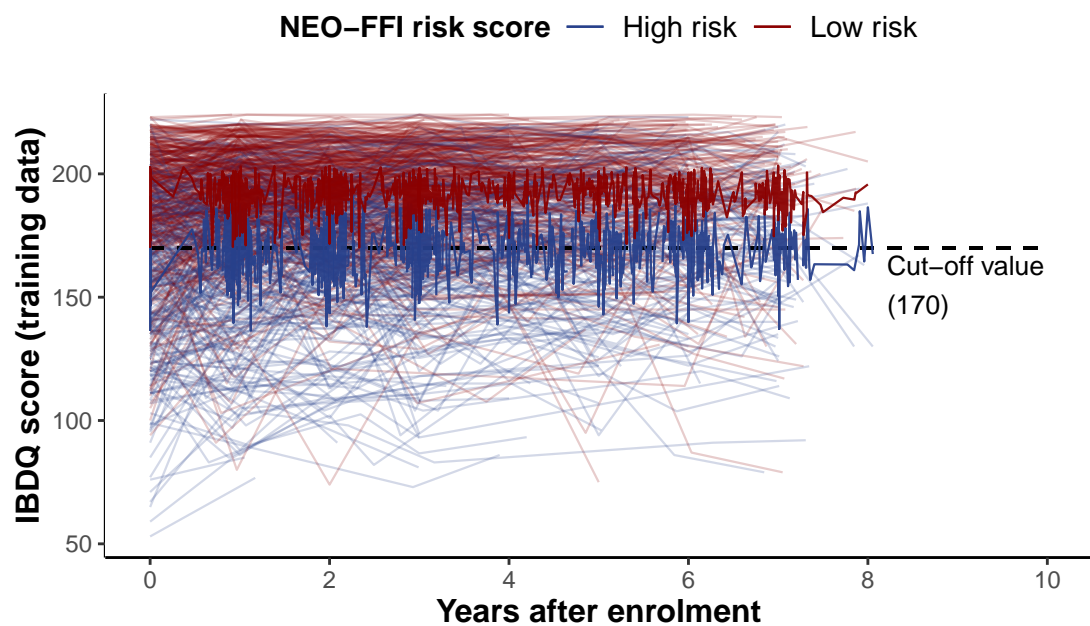**d**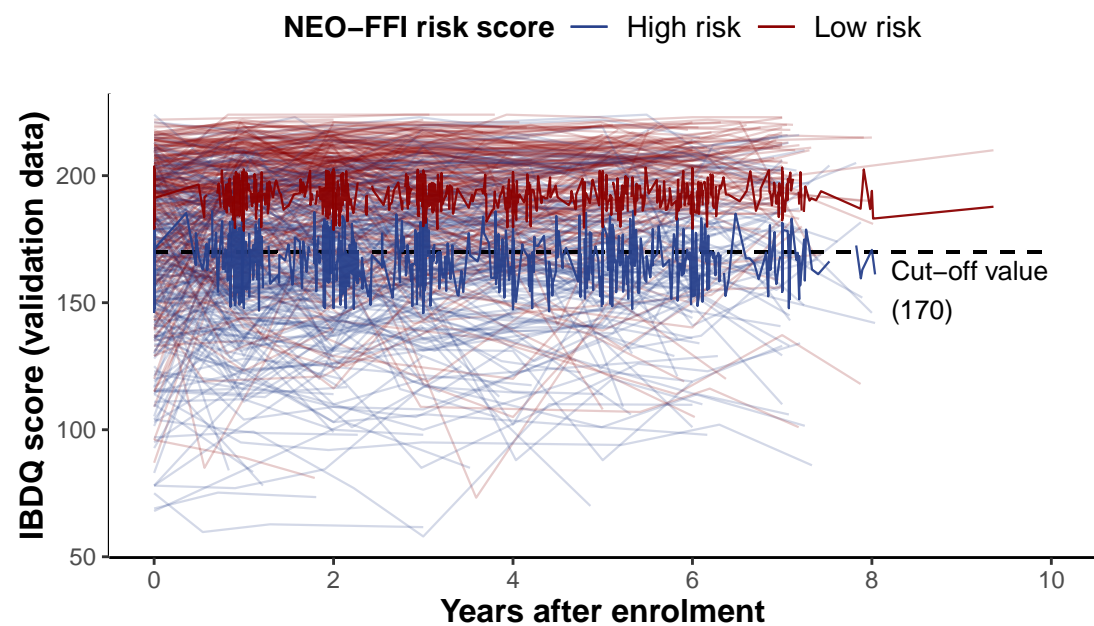

Supplement: Supplementary file 5 — Supplementary file5 (PDF 262 KB) [file 535_2022_1902_MOESM5_ESM.pdf]

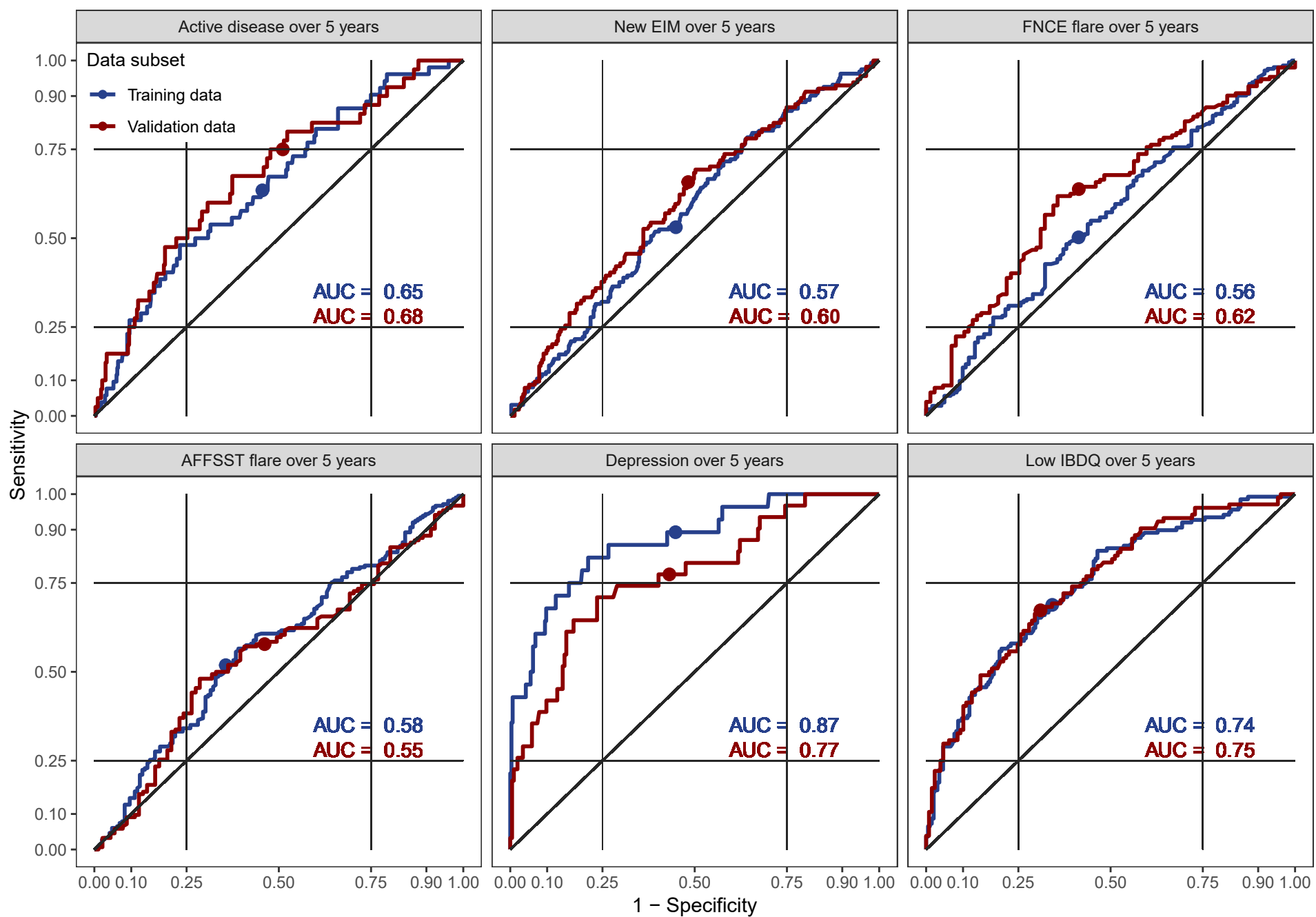

Supplement: Supplementary file 6 — Supplementary file6 (PDF 1591 KB) [file 535_2022_1902_MOESM6_ESM.pdf]

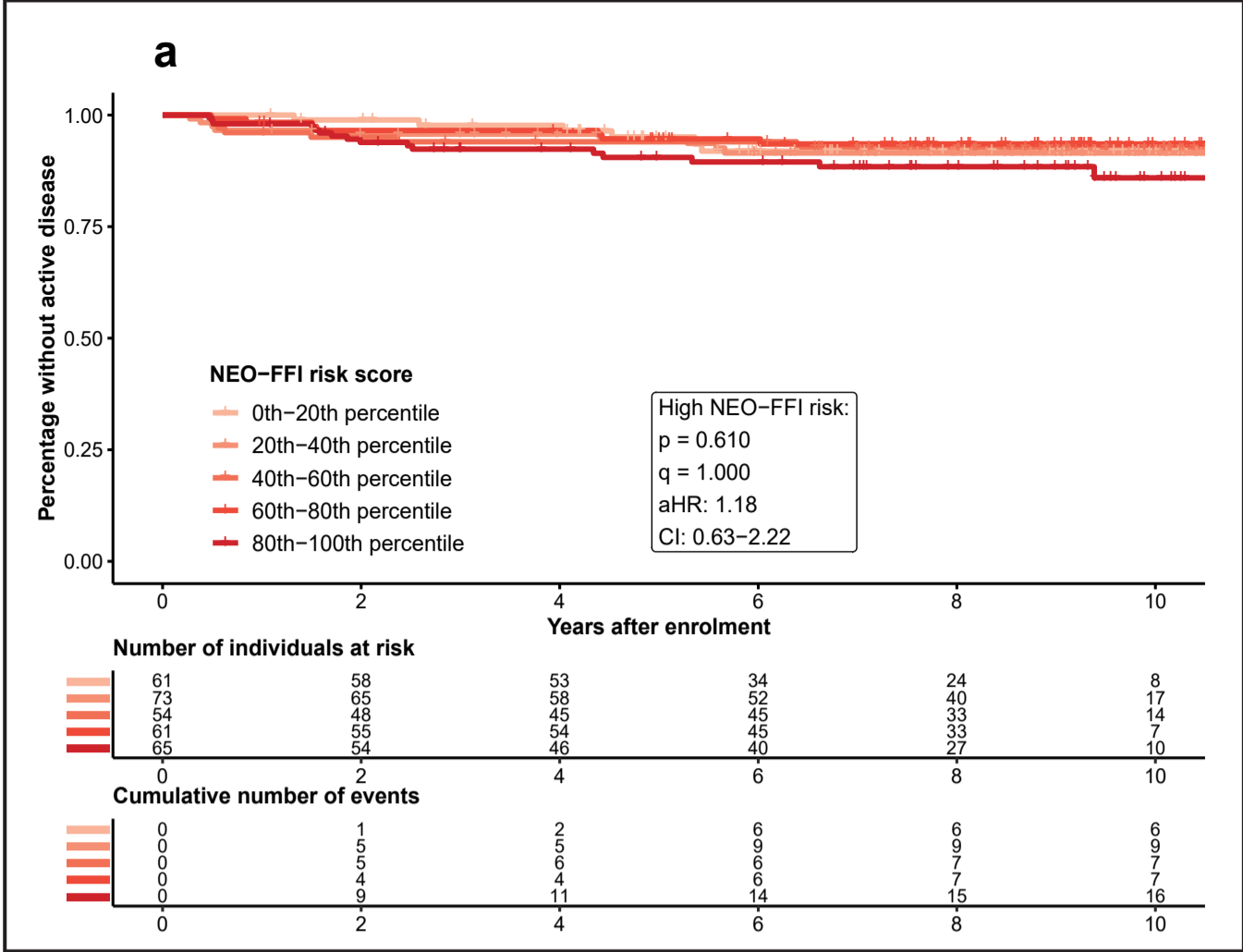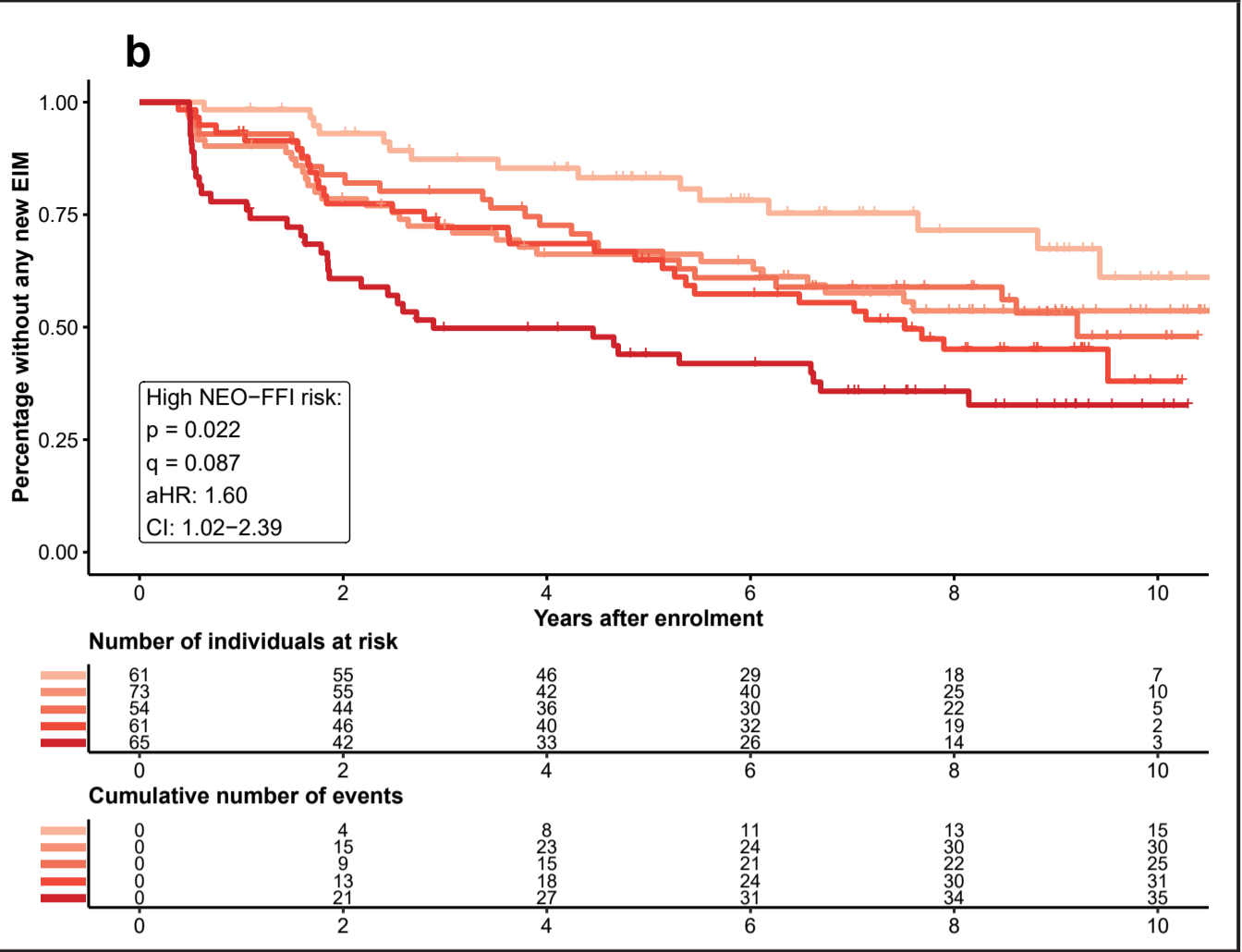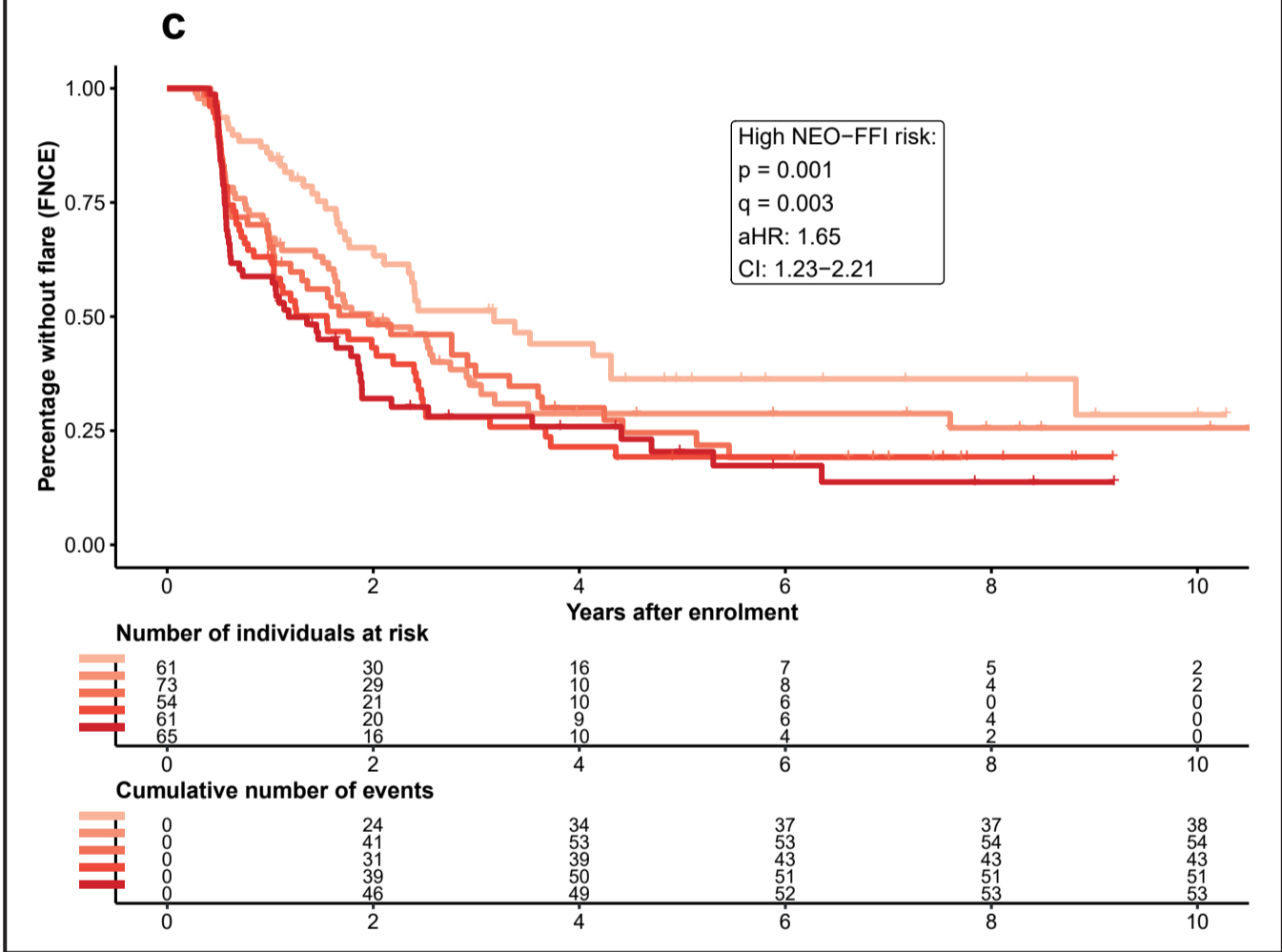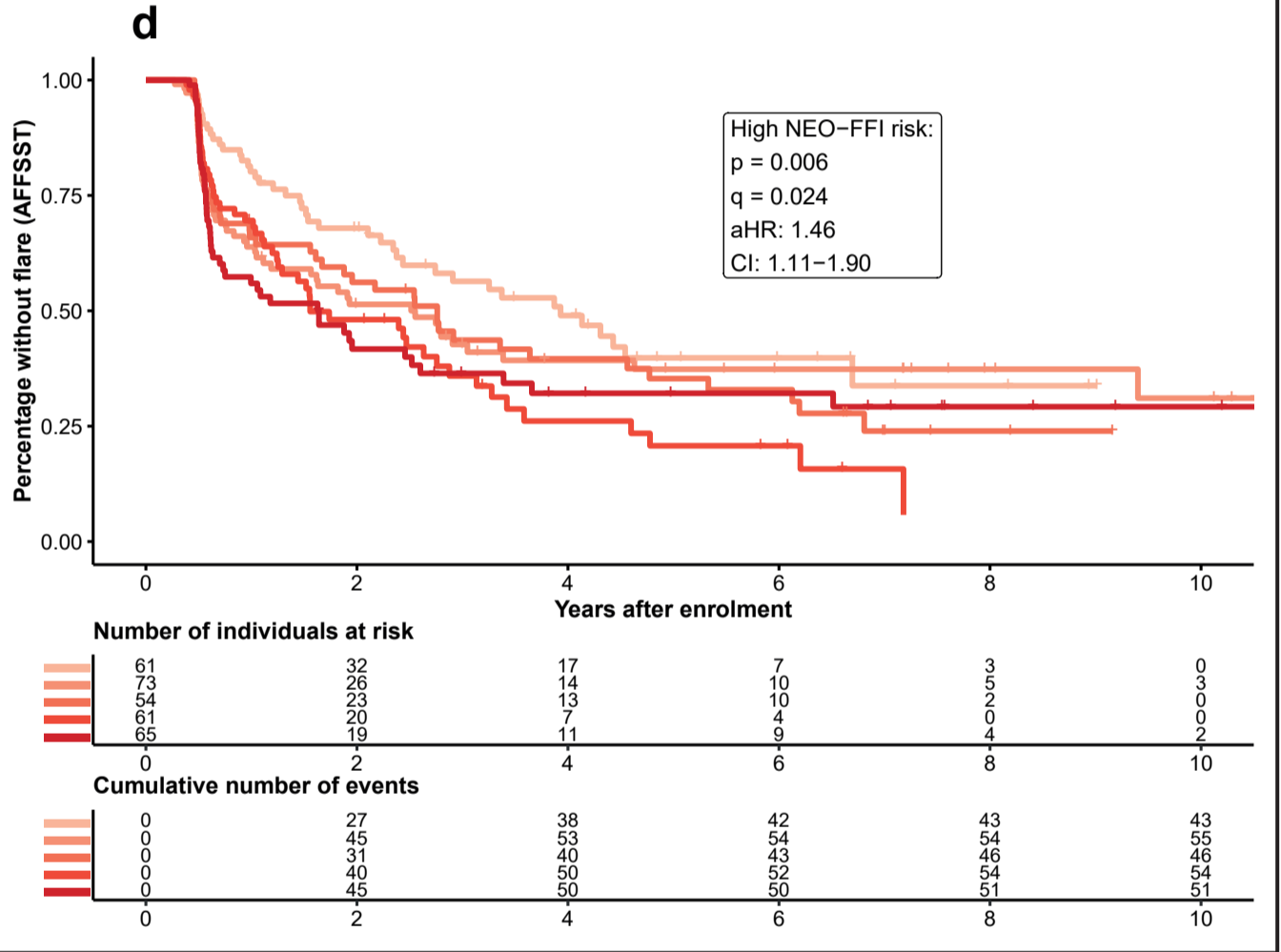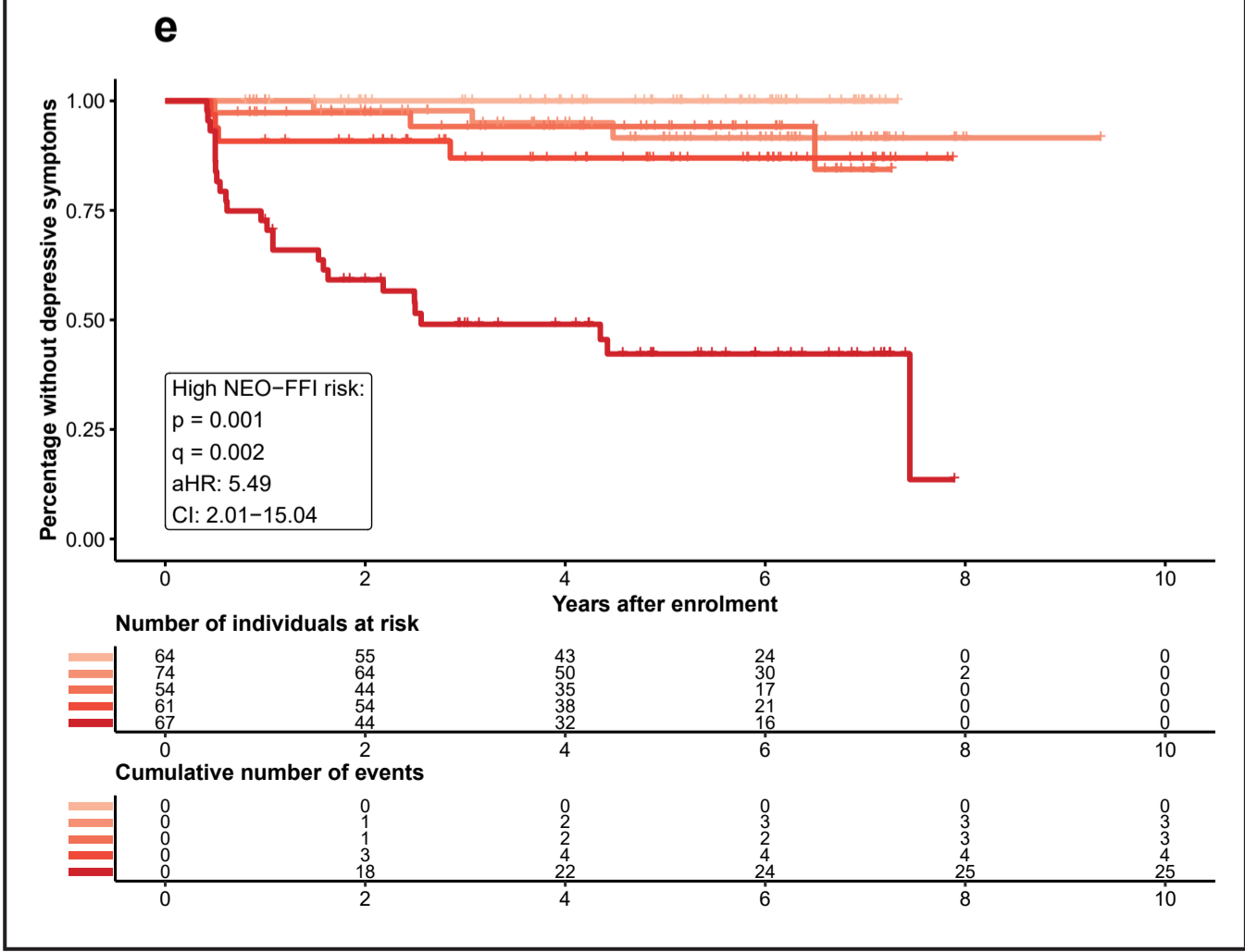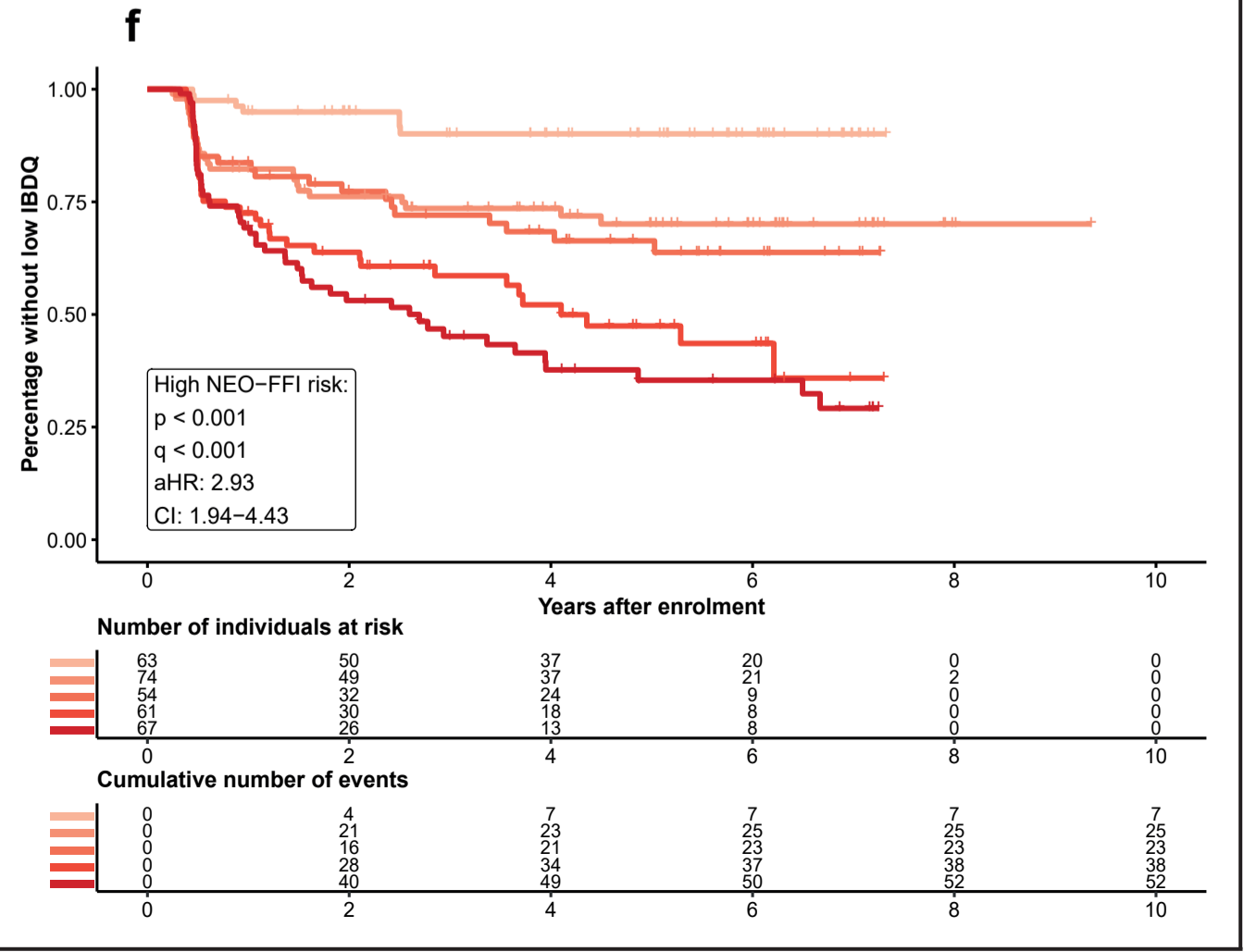

Supplement: Supplementary file 7 — Supplementary file7 (PDF 916 KB) [file 535_2022_1902_MOESM7_ESM.pdf]

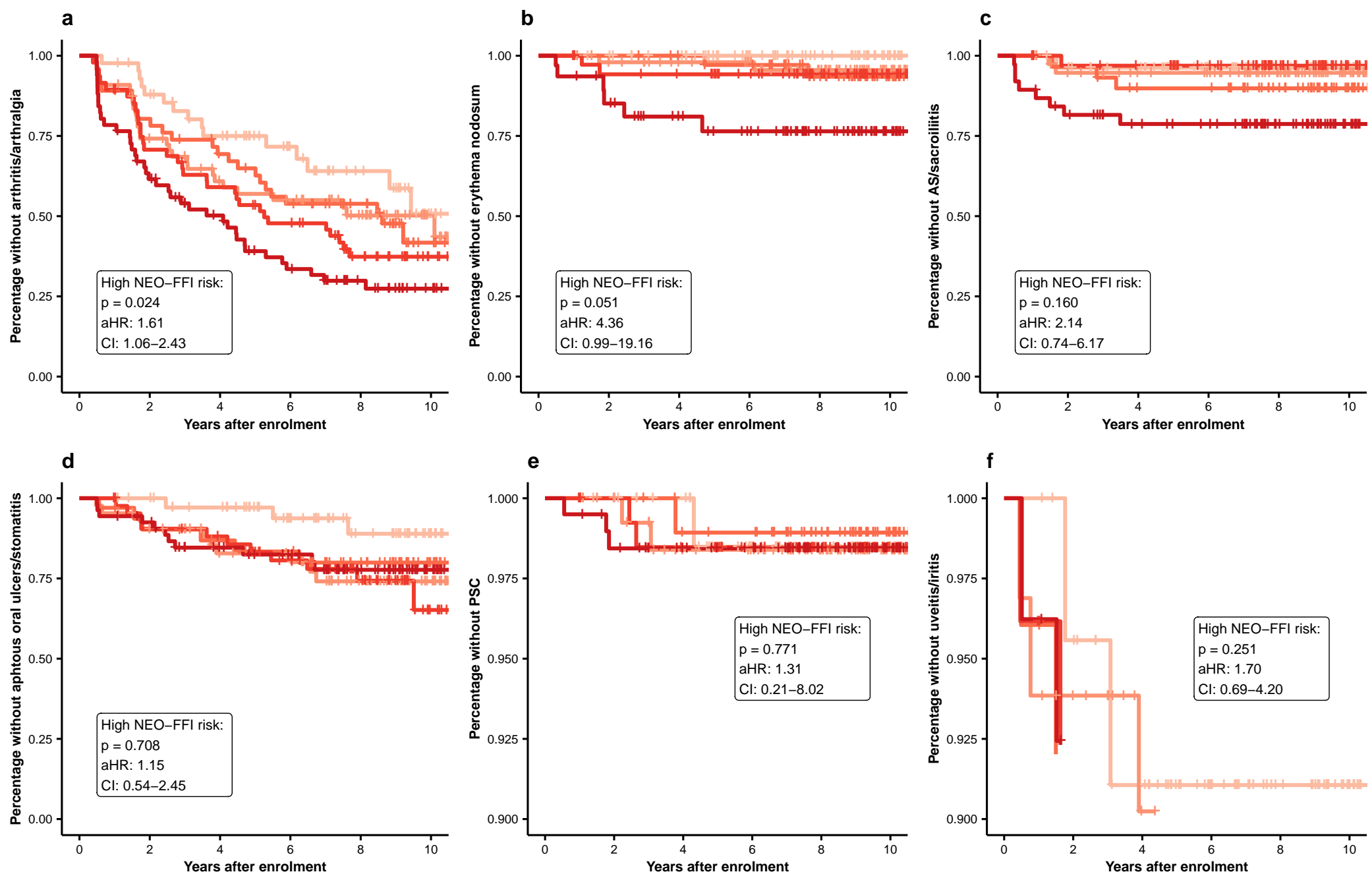

Supplement: Supplementary file 8 — Supplementary file8 (PDF 21 KB) [file 535_2022_1902_MOESM8_ESM.pdf]

**a**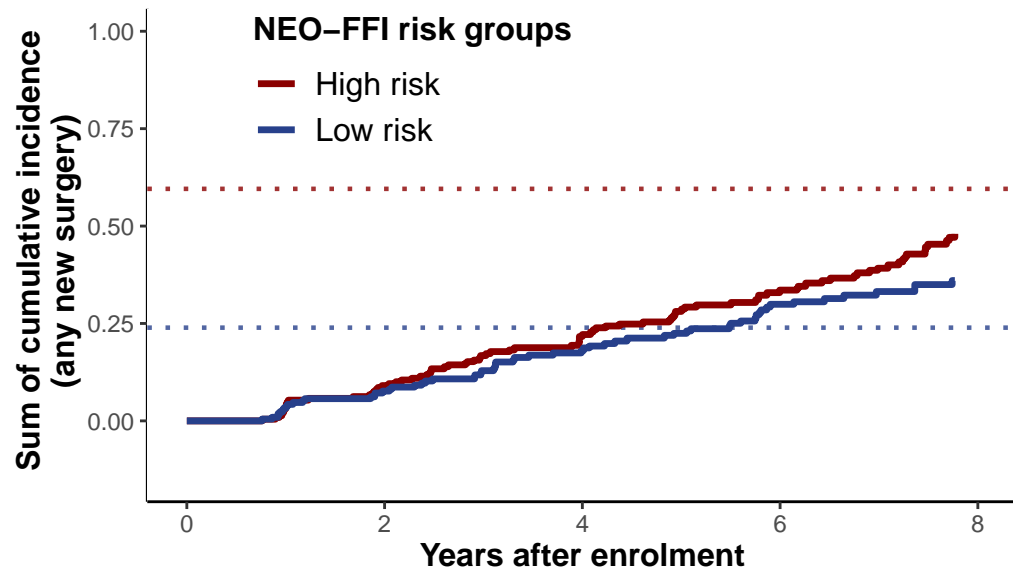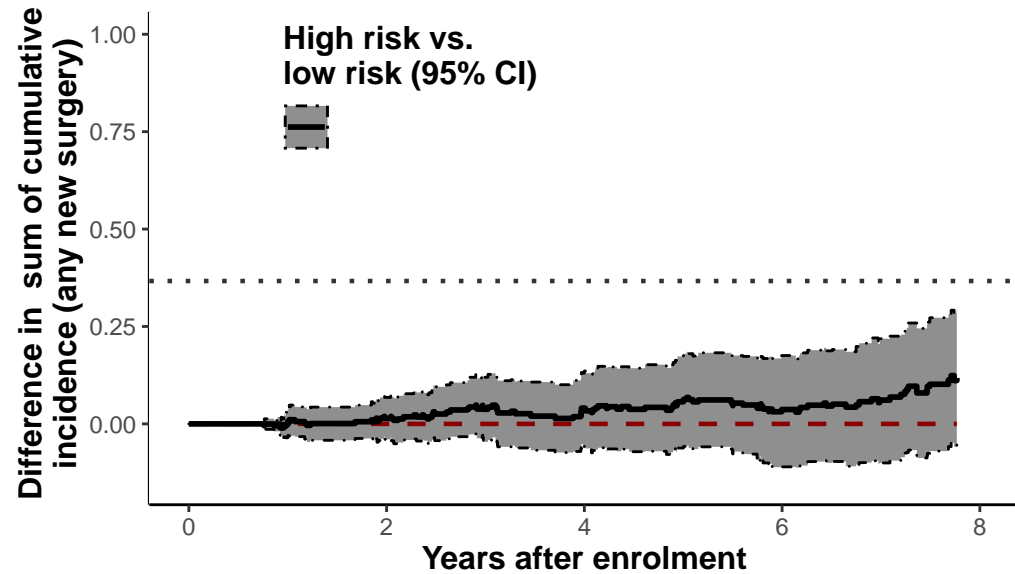

Supplement: Supplementary file 9 — Supplementary file9 (PDF 103 KB) [file 535_2022_1902_MOESM9_ESM.pdf]
