# Supplementary material for: The personality traits activity, self-reproach, and negative affect jointly predict clinical recurrence, depressive symptoms, and low quality of life in inflammatory bowel disease patients
Source: J Gastroenterol. 2022 Jul 28;57(11):848–66. doi: 10.1007/s00535-022-01902-7 (PMC9596530; doi:10.1007/s00535-022-01902-7)
Supplement: Supplementary file 4 — Supplementary file4 (PDF 210 KB) [file 535_2022_1902_MOESM4_ESM.pdf]

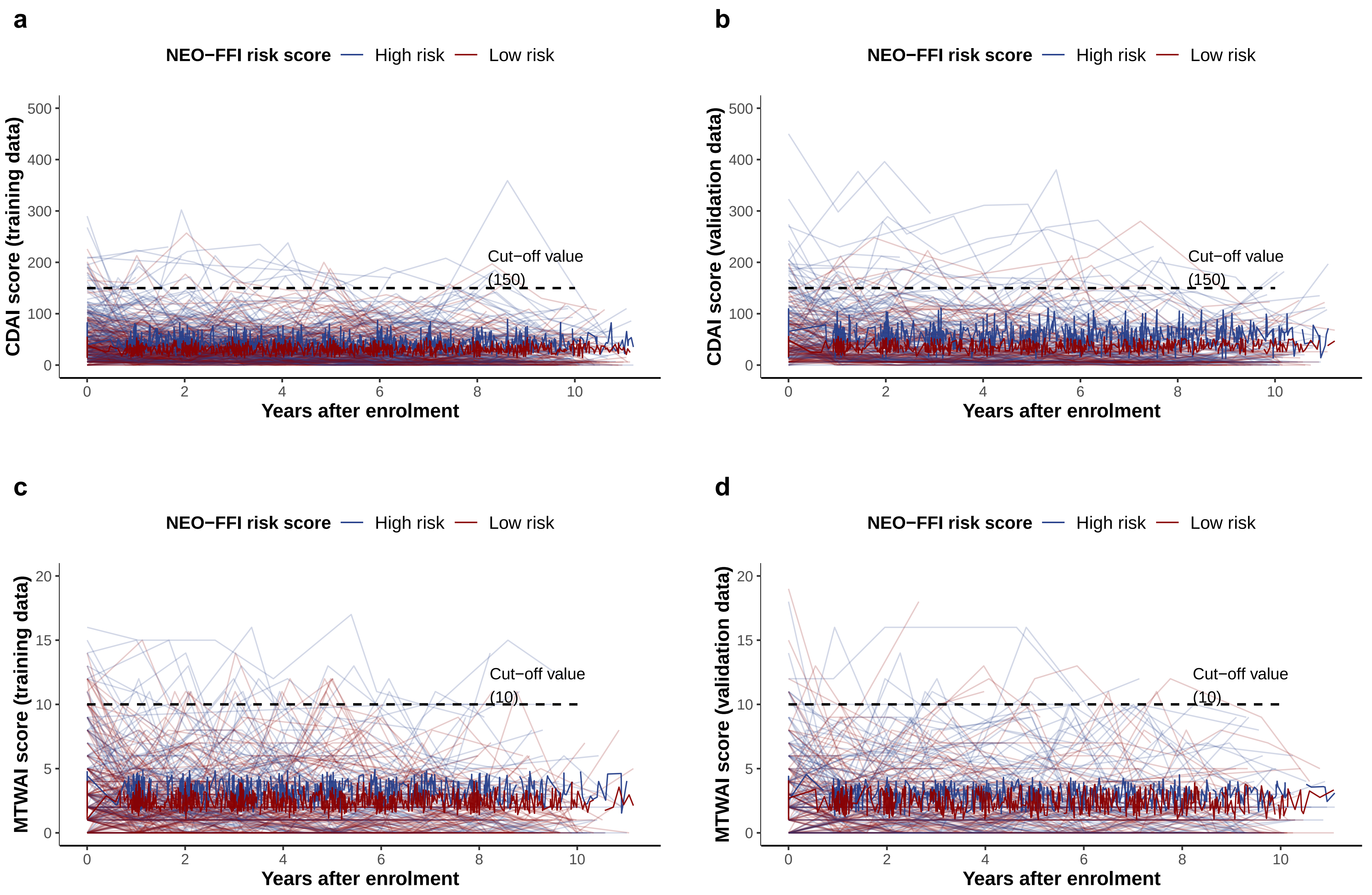

a
NEO−FFI risk score
High risk
Low risk
CDAI score (training data)
Cut−off value
(150)
Years after enrolment
b
NEO−FFI risk score
High risk
Low risk
CDAI score (validation data)
Cut−off value
(150)
Years after enrolment
c
NEO−FFI risk score
High risk
Low risk
MTWAI score (training data)
Cut−off value
(10)
Years after enrolment
d
NEO−FFI risk score
High risk
Low risk
MTWAI score (validation data)
Cut−off value
(10)
Years after enrolment
