# Supplementary material for: The personality traits activity, self-reproach, and negative affect jointly predict clinical recurrence, depressive symptoms, and low quality of life in inflammatory bowel disease patients
Source: J Gastroenterol. 2022 Jul 28;57(11):848–66. doi: 10.1007/s00535-022-01902-7 (PMC9596530; doi:10.1007/s00535-022-01902-7)
Supplement: Supplementary file 10 — Supplementary file10 including all supplementary materials (DOCX 1852 KB) [file 535_2022_1902_MOESM10_ESM.docx]

Supplementary tables

Supplementary table 1

| **Variable** | **Defining criteria (reached upon first occurrence of any of the following)** |
| --- | --- |
| Complications | Colon dysplasia Colorectal cancer Intestinal lymphoma Anaemia (not as an adverse event of medical therapies) Deep vein thrombosis Pulmonary embolism Massive haemorrhage  Nephrolithiasis  Gallstones  Malabsorption syndrome Osteopenia/osteoporosis  Perforation  Peritonitis |
| Extraintestinal manifestations | Peripheral arthritis/ arthralgia Uveitis/iritis  Aphthous oral ulcers/ stomatitis  Erythema nodosum  Pyoderma gangrenosum,  Ankylosing spondylitis/ sacroiliitis PSC |
| Stenosis, localized in | Oesophagus  Duodenum/ jejunum Ileum  Large bowel Rectum Anus |
| IBD-related perianal disease | Fistula Abscesses Anal fissure |
| Surgery | Any abdominal surgery Fistula-related surgery Abscess-related surgery |
| Systemic steroids  (oral steroids with systemic activity) | Prednisone Prednisolone Deflazacort |
| TNF inhibitors | Infliximab,  Certolizumab pegol, Adalimumab  (No patient with golimumab treatment was reported at the time of data export) |
| Number of current therapies | Sum of all administered therapies during the respective time period |
| Smoking status | Smoker, independent from the smoking quantity |
| Academic education | ISCED 2011; level 6-8 |
| Physically active | ≥1 exercise d per week. The number of exercises per week was assessed by a patient questionnaire and included any self-declared physical exercise. |
| **Supplementary table 1 Detailed definition of variables.** Abbreviations: PSC: Primary sclerosing cholangitis, IBD: inflammatory bowel disease, ISCED: International Standard Classification of Education, TNF: tumour necrosis factor | |

Supplementary table 2

| **Domains and cluster subcomponents** | **Items** |
| --- | --- |
| **Neuroticism** |  |
| - Self-reproach | 6, 21, 26, 36, 14, 51, 56 |
| - Negative affect | 1 **^a^**, 11, 16, 31 **^a^**, 46 |
| **Extraversion** |  |
| - Positive affect | 7, 12 **^a^**, 37, 42 **^a^** |
| - Sociability | 2, 17, 27 **^a^**, 57 **^a^** |
| - Activity | 22, 32, 47, 52 |
| **Openness** |  |
| - Aesthetic interests | 13, 23 **^a^**, 43 |
| - Intellectual interests | 48 **^a^**, 53, 58 |
| - Unconventionality | 3 **^a^**, 8 **^a^**, 18 **^a^**, 38 **^a^** |
| **Agreeableness** |  |
| - Non-antagonistic orientation | 9 **^a^**, 14 **^a^**, 19, 24 **^a^**, 29 **^a^**, 44 **^a^**, 54 **^a^**, 59 **^a^** |
| - Prosocial orientation | 4, 34, 39 **^a^**, 49 |
| **Conscientiousness** |  |
| - Orderliness | 5, 10, 15 **^a^**, 30 **^a^**, 55 **^a^** |
| - Goal-striving | 25, 35, 60 |
| - Dependability | 20, 40, 45 **^a^**, 50 |
| **^a^** Reversely keyed item. | |

**Supplementary table 2 Domains and cluster subcomponents of the NEO-FFI.** Summary of the domains and cluster subcomponents (alternative 1) assessed by the NEO-FFI and the corresponding inventory item numbers. Please note that two items (items 28 and 33), belonging to the Openness domain were not assessed by SIBDC questionnaires. Abbreviation: NEO-FFI: NEO Five-Factor Inventory

Supplementary table 3

| \| **NEO-FFI cluster subcomponents** \| **Weighting factors** \| **Rescaling factors** \| \| --- \| --- \| --- \| \| *Activity (reverse)* = A \| 0.055 \| 10 \| \| *Self-reproach* = S \| 0.030 \| 10 \| \| *Negative affect* = N \| 0.043 \| 10 \| \| Formula to calculate the NEO-FFI risk score = NRS \| $x_{NRS} = \left( x_{A}\times0.055 \times10 \right)+\left( x_{S} \times0.030 \times10 \right)+\left( x_{N} \times0.043 \times10 \right)$ \| \| \| **Supplementary table 3 Formula to calculate the NEO-FFI risk score.** Formula and its components to calculate the NEO-FFI risk score for patients. The weighting factors are the corresponding NEO-FFI cluster subcomponents’ bootstrap-derived mean linear regression coefficients from univariable models with disease activity at enrolment as dependent variable (compare Methods). The consistently used rescaling factor = 10 serves only to improve readability of the resulting NEO-FFI risk score values. In the Formula, $x$ stands for a patient’s individual value of the respective NEO-FFI cluster subcomponent or the NEO-FFI risk score, respectively. Abbreviation: NEO-FFI: NEO Five-Factor Inventory \| \| \| |
| --- | --- | --- | --- | --- | --- | --- | --- | --- | --- | --- | --- | --- | --- | --- | --- | --- | --- | --- |

Supplementary table 4

| Dependent variable | **Neuroticism** | **Extraversion** | **Openness** (items 28 and 33 missing) ^a^ | **Agreeable-ness** | **Conscientious-ness** | **NEO-FFI(-R) risk score** |
| --- | --- | --- | --- | --- | --- | --- |
| **IBD patient (yes) ^b^** | 0.63  (CI: -0.05-1.32)  *p* = 0.070 | -2.16  (CI: -2.73-(-)1.59)  ***p* < 0.001** | 2.01  (CI: 1.63- 2.39)  ***p* < 0.001** | -0.67  (CI: -1.14-(-)0.20)  ***p* = 0.005** | -0.04  (CI: -0.56-0.48)  *p* = 0.890 | 0.14  (CI: -0.12-0.41)  *p* = 0.289 |
| **Sex (male)** | -2.74  (CI: -3.27-(-)2.20)  ***p* < 0.001** | -0.28  (CI: -0.73-0.16)  *p* = 0.209 | -0.28  (CI: -0.58-0.02)  *p* = 0.065 | -2.18  (CI: -2.55-(-)1.82)  ***p* < 0.001** | 0.22  (CI: -0.19 -0.62)  *p* = 0.299 | -0.94  (CI: -1.15-(-)0.73)  ***p* < 0.001** |
| **Age (years)** | -0.04  (CI: -0.06-(-)0.01)  ***p* = 0.002** | -0.06  (CI: -0.08-(-)0.04)  ***p* < 0.001** | (-)<0.01  (CI: -0.01-0.01)  *p* = 0.978 | - 1. (CI: (-)<0.01-0.03)   *p* = 0.161 | -0.01  (CI: -0.03-0.01)  *p* = 0.278 | (-)<0.01  (CI: -0.01-0.01)  *p* = 0.773 |
| **BMI (kg/m^2^)** | <0.01  (CI: -0.06-0.06)  *p* = 0.940 | <0.01  (CI: -0.05-0.05)  *p* = 0.981 | -0.06  (CI: -0.10-(-)0.03)  ***p* < 0.001** | -0.04  (CI: -0.08-(-)<0.01)  ***p* = 0.034** | -0.07  (CI: -0.11-(-)0.02)  ***p* = 0.004** | 0.01  (CI: -0.01-0.03)  *p* = 0.359 |
| **Smoker (yes)** | 0.77  (CI: -0.16-1.38)  ***p* = 0.014** | 0.16  (CI: -0.36-0.65)  *p* = 0.572 | 0.05  (CI: -0.29-0.40)  *p* = 0.753 | -0.67  (CI: -1.09-(-)0.25)  ***p* = 0.002** | -0.46  (CI: -0.92-0.01)  *p* = 0.055 | 0.21  (CI: -0.03-0.46)  *p* = 0.080 |
| **Daily alcohol consumption (yes)** | 0.13  (CI: -0.52-0.77)  *p* = 0.700 | -0.06  (CI: -0.47-0.59)  *p* = 0.828 | -0.10  (CI: -0.25-0.46)  *p* = 0.567 | -0.52  (CI: -0.96-(-)0.08)  ***p* = 0.020** | -0.69  (CI: -1.18-(-)0.20)  ***p* = 0.005** | 0.18  (CI: 0.07-0.44)  *p* = 0.155 |
| **Academic education (yes)** | -0.88  (CI: -1.49-(-)0.27)  ***p* = 0.005** | 0.81  (CI: 0.31-1.31)  ***p* = 0.002** | 2.94  (CI: 2.60-3.28)  ***p* < 0.001** | -0.69  (CI: -1.10-(-)0.27)  ***p* = 0.001** | -0.38  (CI: -0.84-0.08)  *p* = 0.103 | -0.45  (CI: -0.68-(-)0.21) ***p* < 0.001** |
| **Goodness of fit: *R*^2^/ adjusted *R*^2^** | 0.045 0.043 | 0.024 0.022 | 0.117 0.115 | 0.064 0.062 | 0.008 0.006 | 0.031 0.029 |
| **IBD status’ (IBD patient: yes) effect in maximal score percent ^c^** | +1.3%  (CI: -0.1%-(+)2.7%) | -4.5%  (CI: -5.7%-(-)3.3%) | +5.0%  (CI: +4.1% -(+)6.0%) | -1.2%  (CI: -2.4%-(-)0.4%) | -0.1%  (CI: -1.2%-(+)1.0%) | -0.6% (CI: -0.5%-(+)1.6%) |
| ^a^ NEO-FFI(-R) items 28 and 33 are missing or being dropped, respectively, because they were not assessed in the SIBDCS (compare limitations) ^b^ The independent variable *IBD patient* was coded *yes* for patients of the Swiss IBD Cohort Study and *no* for participants of the CoLaus¦PsyCoLaus study. ^c^ Regression estimate (IBD patient: yes) divided by the maximal possible score value | | | | | | |
| **Supplementary table 4 Personality domains and NEO-FFI(-R) risk score of IBD patients and population-based controls in linear regression models.** Personality domains or NEO-FFI(-R) risk scores were dependent variables. Linear regression coefficients, the corresponding confidence intervals and *p*-values are reported.  Abbreviations: BMI: body mass index, CI: confidence interval, IBD: Inflammatory bowel disease, NEO-FFI(-R): (Revised) NEO-Five-Factor Inventory, *R^2^*: square of the correlation coefficient | | | | | | |

Supplementary table 5

| Dependent variable | **Type D personality (yes)** | | |
| --- | --- | --- | --- |
| Model type | Univariable N=3679 | Multivariable N=3554 | Reduced  N=3526 |
| **IBD patient (yes) ^a^** | 2.41  (CI: 2.04-2.85)  ***p* < 0.001** | 1.86  (CI: 1.49-2.32)  ***p* < 0.001** | 1.84  (CI: 1.49-2.26)  ***p* < 0.001** |
| **Sex (male)** |  | 0.95  (CI: 0.79-1.13)  *p* = 0.545 | Eliminated |
| **Age (years)** |  | 0.98  (CI: 0.98-0.99)  ***p* < 0.001** | 0.98  (CI: 0.98-0.99)  ***p* < 0.001** |
| **BMI (kg/m^2^)** |  | 1.01  (CI: 0.99-1.03)  *p* = 0.568 | Eliminated |
| **Smoker (yes)** |  | 0.95  (CI: 0.78-1.17)  *p* = 0.656 | Eliminated |
| **Daily alcohol consumption (yes)** |  | 1.01  (CI: 0.80-1.27)  *p* = 0.916 | Eliminated |
| **Academic education (yes)** |  | 1.06  (CI: 0.86-1.30)  *p* = 0.587 | Eliminated |
| **Goodness of fit: *McFadden’s R*^2^ / adjusted *McFadden’s R*^2^** | 0.028 / 0.027 | 0.033 / 0.029 | 0.034 / 0.032 |
| **^a^** The independent variable *IBD patient* was coded as *yes* for patients of the Swiss IBD Cohort Study and *no* for participants of the CoLaus¦PsyCoLaus study. | | | |
| **Supplementary table 5 Predictors for Type D personality in SIBDC patients and population-based controls.** Logistic regression models with Type D personality (yes/no) as dependent variable are presented. 3 models were fitted: a univariable model, a complete multivariable model including potential confounders and a reduced model, obtained by optimizing the Akaike information criterion. Odds ratios, the corresponding confidence intervals and *p*-values are reported.  Abbreviations: IBD: Inflammatory bowel disease, BMI: body mass index, NEO-FFI(-R): (Revised) NEO-Five-Factor Inventory | | | |

Supplementary figures

Supplementary figure 1


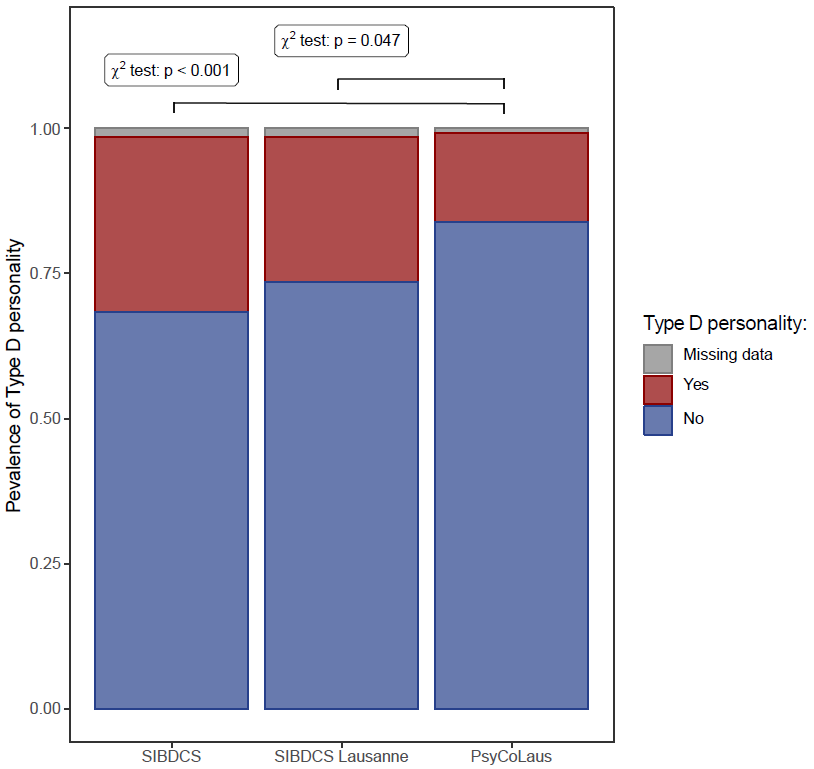


**Supplementary figure 1 Bar plot illustrating higher prevalence of Type D personality amongst IBD patients.** **Analyses**: *χ*^2^ tests. **Abbreviations**: NEO-FFI: NEO Five-Factor Inventory, *p*: *p*-value, SIBDCS: Swiss IBD Cohort Study

Supplementary figure 2


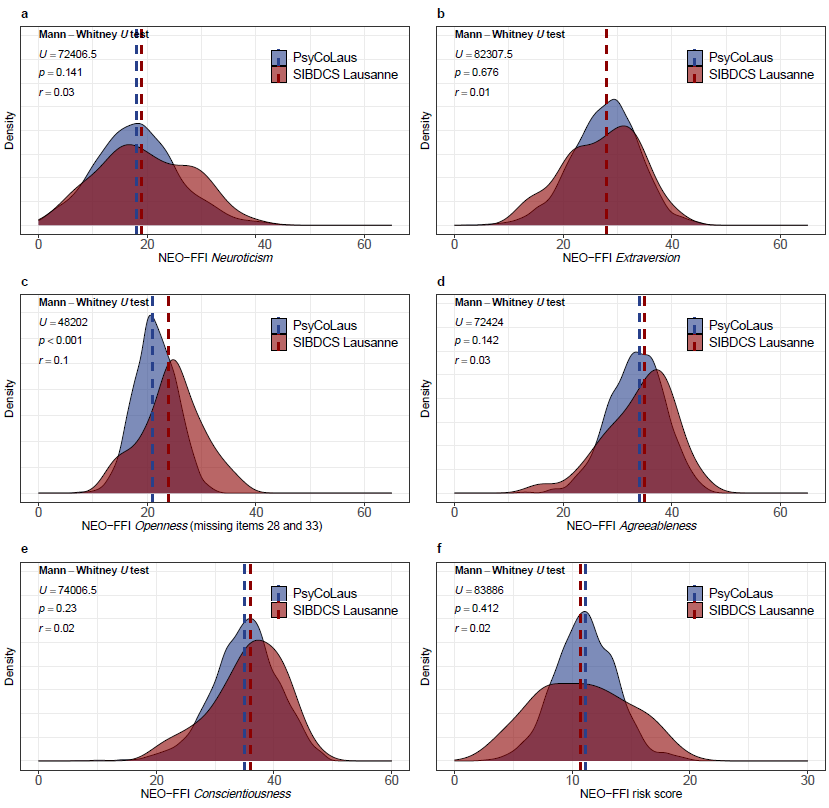


**Supplementary figure 2: Sensitivity analysis comparing personality profiles from Lausanne SIBDC patients with CoLaus¦PsyCoLaus participants.** Density plots for the personality domains *Neuroticism* (**a**), *Extraversion* (**b**), *Openness* (**c**), *Agreeableness* (**d**) and *Conscientiousness* (**e**) as well as for the NEO-FFI risk score (**f**) stratified for the indicated cohorts. Only SIBDCS patients living in Lausanne were included for direct comparison with CoLaus¦PsyCoLaus participants (all residing in Lausanne). Number of SIBDCS patients for analysis of Neuroticism: N=62, Extraversion: N=63, Openness: N=61, Agreeableness: N=64, Conscientiousness: N=64 and NEO-FFI risk score: N=64. Red/blue dashed lines indicate the respective cohort’s median score value. **Analyses**: Mann-Whitney *U* test. **Abbreviations**: NEO-FFI: NEO Five-Factor Inventory, *p*: *p*-value, r: Pearson's *r*, SIBDCS: Swiss IBD Cohort Study, *U*: *U* statistic

Supplementary figure 3


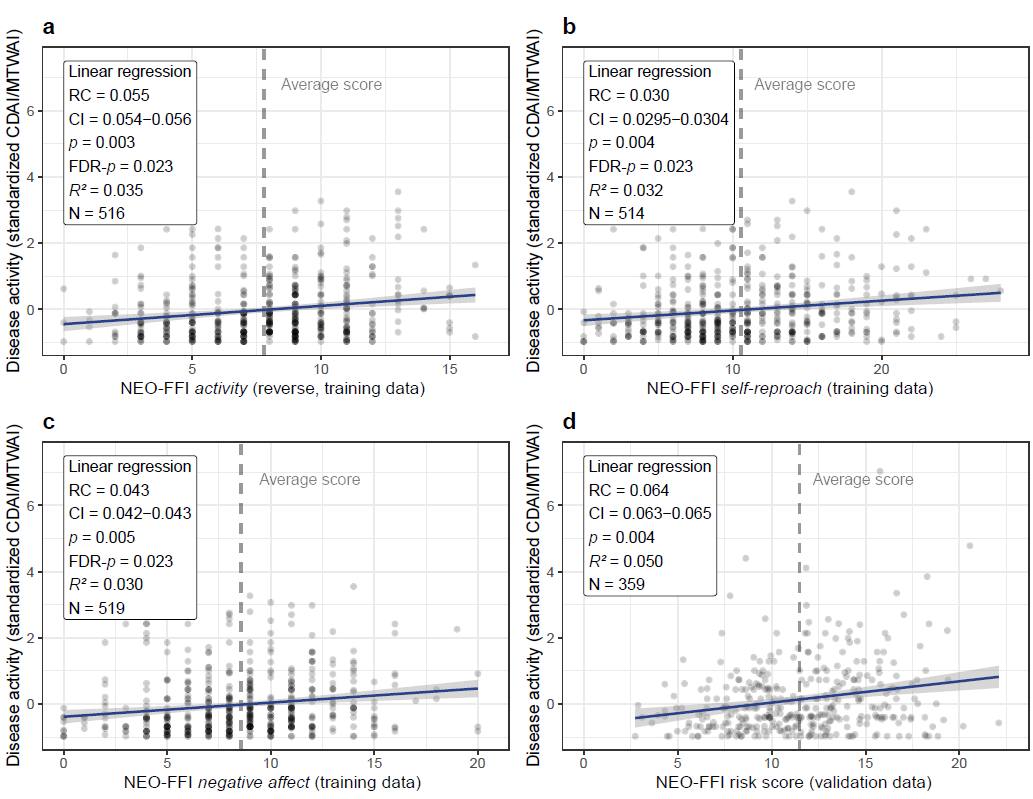


**Supplementary figure 3 NEO-FFI subdomains and the NEO-FFI risk score associated with disease activity.** **A-C:** Personality subcomponents identified by screening for association with disease activity in the training data set; **A**: NEO-FFI *activity* scores (reverse), **B:** NEO-FFI *self-reproach* scores, **C:** NEO-FFI *negative affect* scores. **D:** Confirming an association of the NEO-FFI risk score with disease activity levels in the validation data set. Regression lines (blue) with 95% CI are indicated. Abbreviations: CDAI: Crohn's Disease Activity Index, MTWAI: Modified Truelove and Witts Severity Index, N: number of patients, NEO-FFI: NEO Five-Factor Inventory, *p* = *p*-value, FDR-*p*: FDR-controlled *p*-value, *R^2^*: square of the correlation coefficient, RC: regression coefficient, CI: 95% confidence interval

Supplementary figure 4


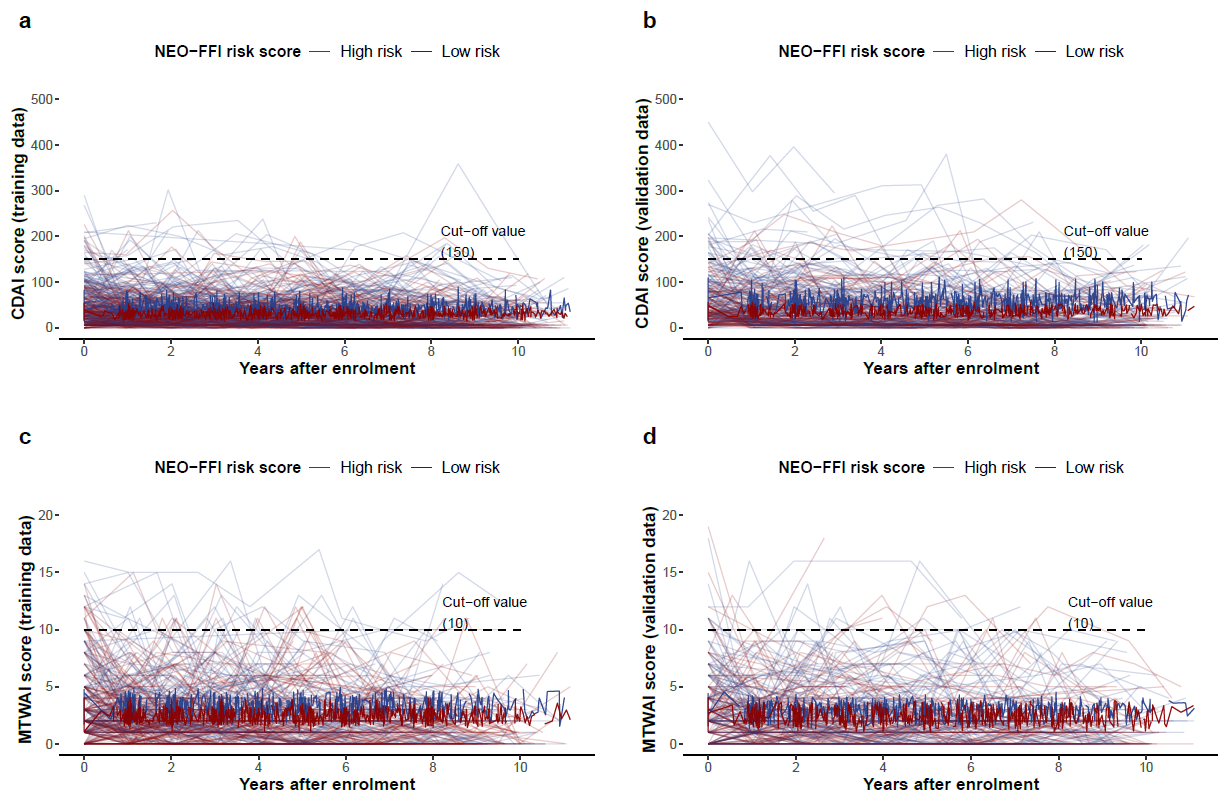


**Supplementary figure 4 Variation of disease activity scores over time.** Fluctuations lines of individual patients (thin and coloured lines) for disease activity scores stratified for high and low NEO-FFI risk scores. Dashed horizontal lines mark the sore values used for dichotomisation of the respective score in this study. Bold highlighted and coloured (red/blue) indicate moving averages of the respective NEO-FFI risk group. Abbreviations: CDAI: Crohn's Disease Activity Index, MTWAI: Modified Truelove and Witts Severity Index, NEO-FFI: NEO Five-Factor Inventory, CI: confidence interval

Supplementary figure 5


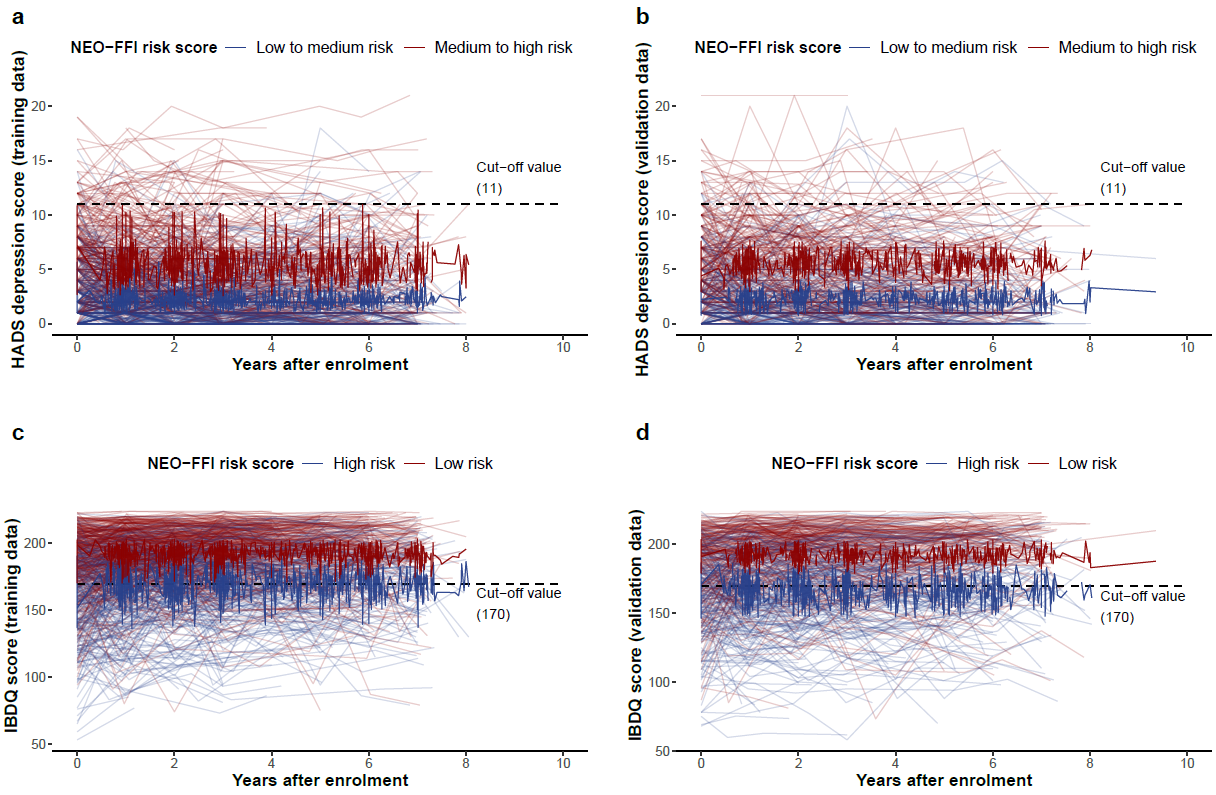


**Supplementary figure 5 Variation of psychological scores over time.** Fluctuations lines of individual patients (thin and coloured lines) for psychological scores stratified for high and low NEO-FFI risk. Dashed horizontal lines mark the sore values used for dichotomisation of the respective score in this study. Bold highlighted and coloured (red/blue) indicate moving averages of the respective NEO-FFI risk group. Abbreviations: HADS: Hospital Anxiety and Depression Scale, IBDQ: Inflammatory Bowel Disease Questionnaire, NEO-FFI: NEO Five-Factor Inventory, CI: confidence interval

Supplementary figure 6


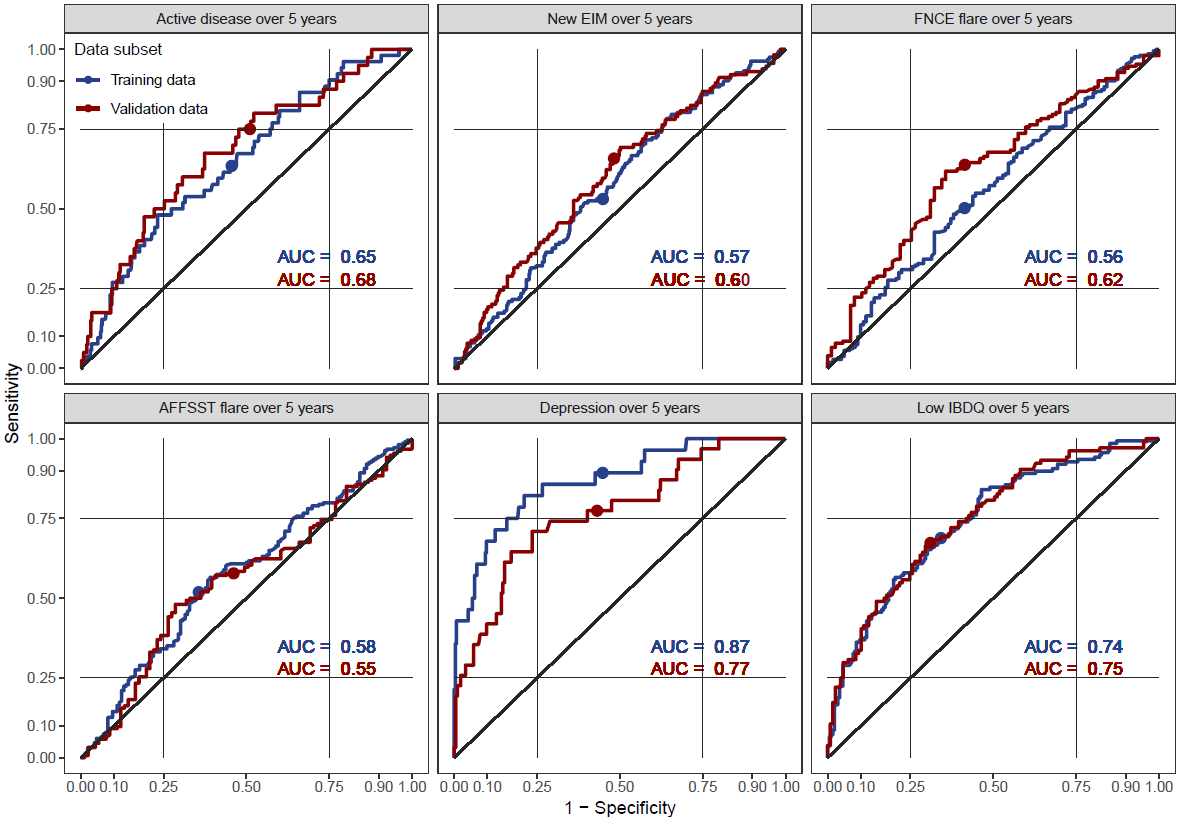


**Supplementary figure 6** **Receiver operating characteristic curves for the NEO-FFI risk score in the training and validation data set.** Position of the cut-off for the NEO-FFI risk score used for dichotomisation (11.3) is marked every curve as a dot together with the area under the curve (AUC) value.

Supplementary figure 7


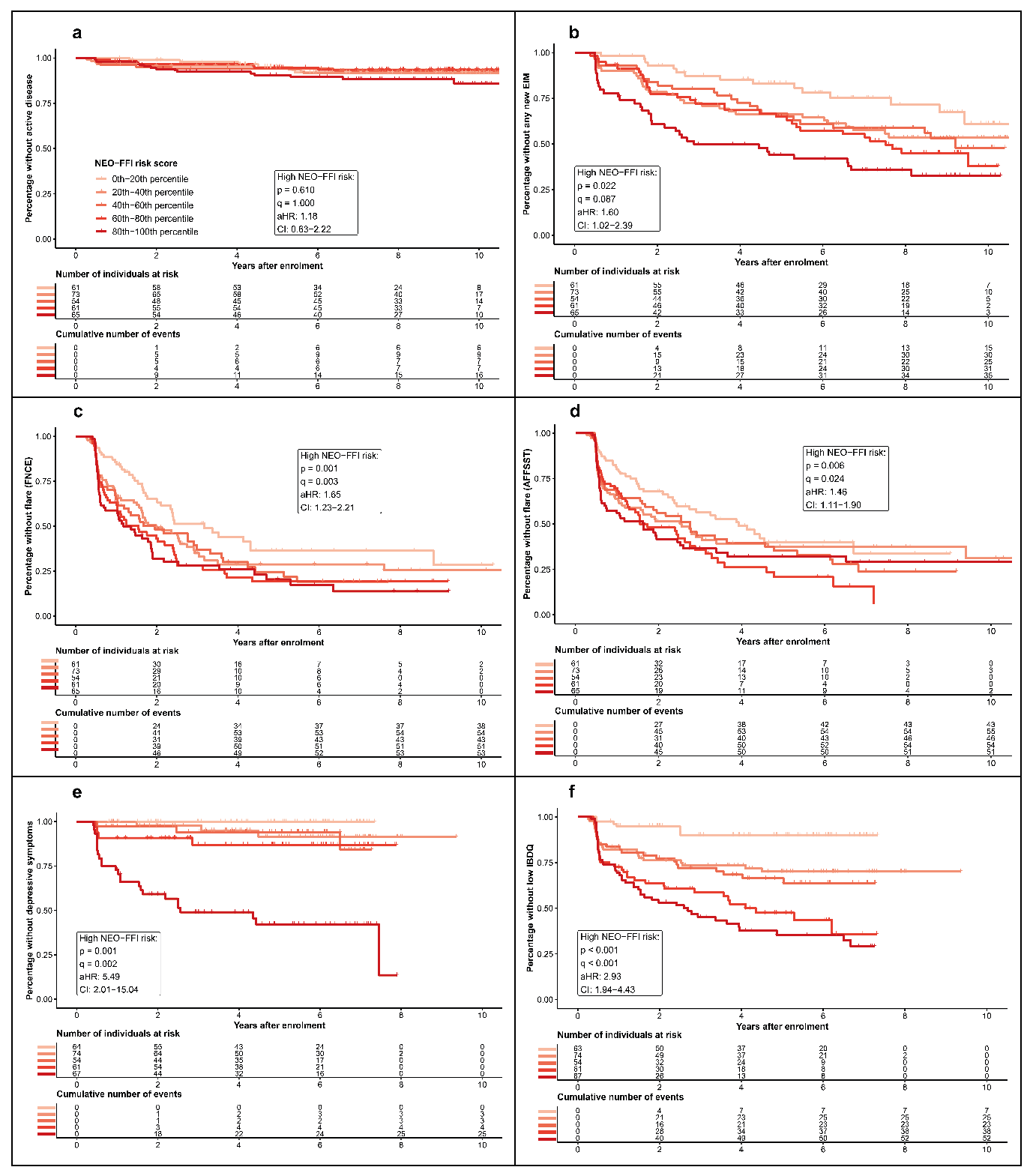


**Supplementary figure 7 High NEO-FFI risk increases the hazards for clinical deterioration in patients in remission.** Kaplan Meier curves for patients in remission (CDAI<150/MTWAI<10) and corrected for confounders including Type D personality (see methods) for active disease (**a**), new EIMs (**b**) FNCE flares (**c**), AFFSST flares (**d**), depressive symptoms (**e**) and low IBDQ (**f**) are presented. For the graphics, IBD patients in remission are stratified for 5 groups according to NEO-FFI risk score 20 percentile intervals. The estimates describe the comparison of the high NEO-FFI risk group with the low NEO-FFI risk group (cut-off 11.3). Depressive symptoms were defined as HADS-D≥11. Low IBDQ was defined as values below 170. **Analyses**: multivariable Cox proportional hazards models. **Abbreviations**: AFFSST: active disease, physician reported flare, new fistula, new stenosis, surgery, or new systemic therapy, aHR: adjusted hazard ratio, CDAI: Crohn's Disease Activity Index, CI: confidence interval, EIM: extraintestinal manifestation, FNCE: physician reported flare, non-response to therapy, HADS: Hospital Anxiety and Depression Scale, IBD: inflammatory bowel disease, IBDQ: Inflammatory Bowel Disease Questionnaire, MTWAI: Modified Truelove and Witts Severity Index, NEO-FFI: NEO Five-Factor Inventory.

Supplementary figure 8


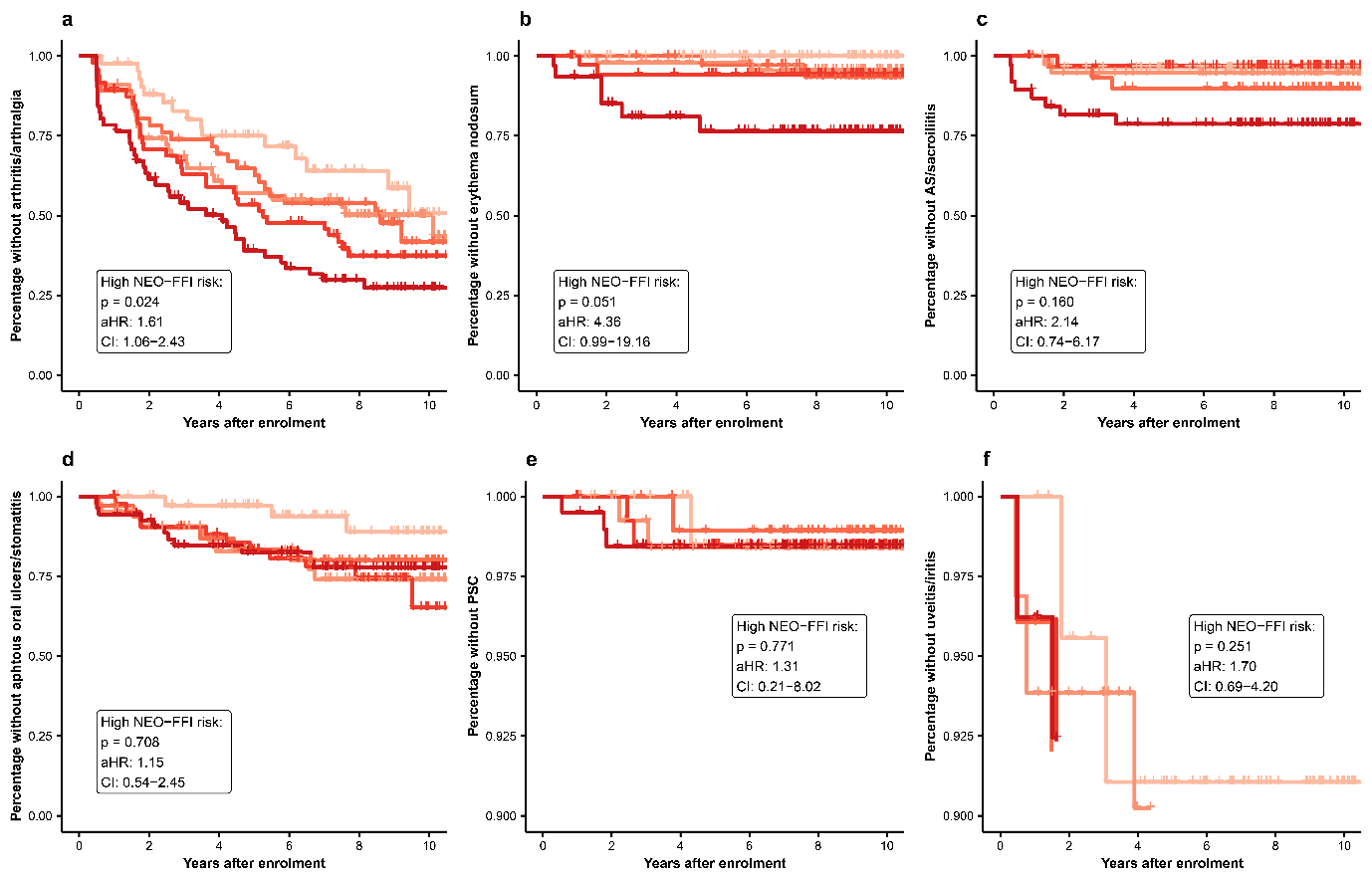


**Supplementary figure 8 High NEO-FFI risk tends to increase the hazards for new occurrence of individual EIMs in IBD patients.** Kaplan Meier curves corrected for confounders including Type D personality (see methods) for the new occurrence of individual EIMs (**a-f**). For the graphics, IBD patients are stratified for 5 groups according to NEO-FFI risk score 20 percentile intervals. The estimates describe the comparison of the high NEO-FFI risk group with the low NEO-FFI risk group (cut-off 11.3). Due to rare occurrence rates of the respective EIMs the y-axis scale for panels **e** and **f** were adjusted to improve visualisation. **Analyses**: multivariable Cox proportional hazards models. **Abbreviations**: AFFSST: active disease, physician reported flare, new fistula, new stenosis, surgery, or new systemic therapy, aHR: adjusted hazard ratio, AS: ankylosing spondylitis, CDAI: Crohn's Disease Activity Index, CI: confidence interval, EIM: extraintestinal manifestation, FNCE: physician reported flare, non-response to therapy, HADS: Hospital Anxiety and Depression Scale, IBD: inflammatory bowel disease, IBDQ: Inflammatory Bowel Disease Questionnaire, MTWAI: Modified Truelove and Witts Severity Index, NEO-FFI: NEO Five-Factor Inventory, PSC: primary sclerosing cholangitis.

Supplementary figure 9


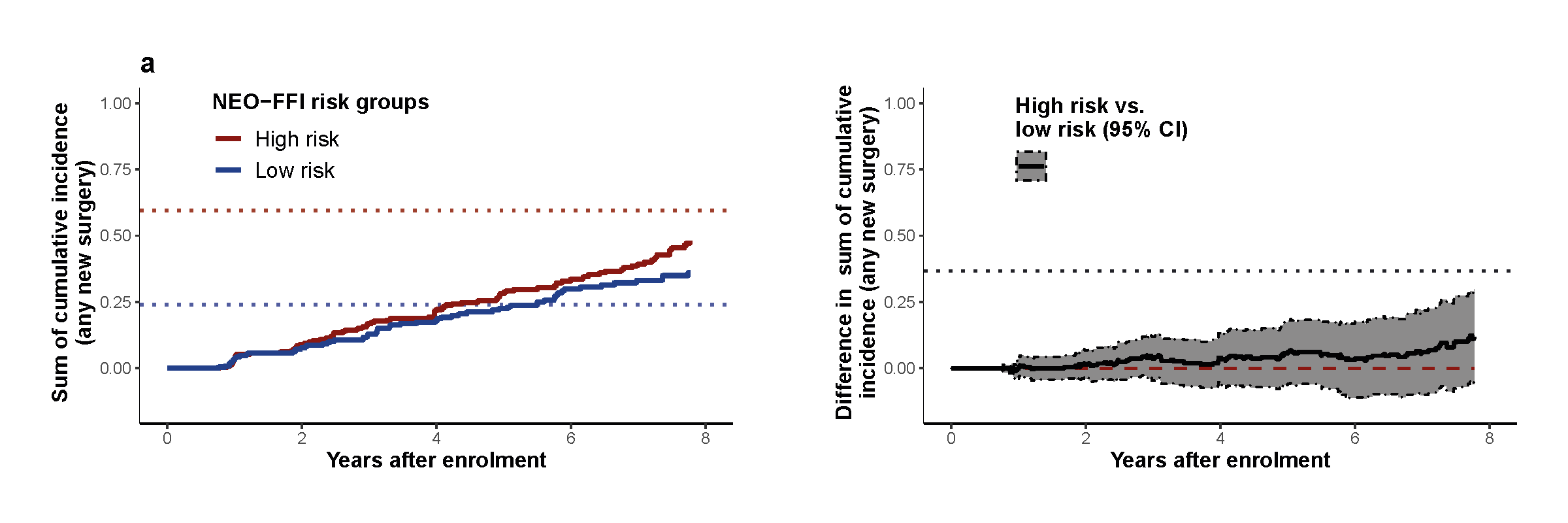


**Supplementary figure 9 High NEO-FFI risk non-significantly increases the cumulative number of surgeries.** Plots illustrating the cumulative incidence counts (left) and the differences of cumulative incidence between low and high NEO-FFI risk groups (right) for disease related surgeries in IBD patients. Dotted lines in the left plots indicate the maximal values observed and the red dashed line in the right plots mark the zero-difference line. Overlap of the zero-difference line and the 95% confidence interval indicates lack of significance. Results were obtained by bootstrapping with 1000-fold resampling. **Analyses**: cumulative incidence analyses. **Abbreviations**: CI: confidence interval, IBD: inflammatory bowel disease, NEO-FFI: NEO Five-Factor Inventory.
